# Central data monitoring in the multicentre randomised SafeBoosC-III trial – a pragmatic approach

**DOI:** 10.1186/s12874-021-01344-4

**Published:** 2021-07-31

**Authors:** Markus Harboe Olsen, Mathias Lühr Hansen, Sanam Safi, Janus Christian Jakobsen, Gorm Greisen, Christian Gluud, The SafeBoosC-III Trial Group, Adelina Pellicer, Agata Bargiel, Andrew Hopper, Anita Truttmann, Anja Klamer, Anne Marie Heuchan, Asli Memisoglu, Barbara Krolak-Olejnik, Beata Rzepecka, Bergona Loureiro, Chantal Lecart, Cornelia Hagmann, Ebru Ergenekon, Eleftheria Hatzidaki, Emmanuele Mastretta, Eugene Dempsey, Evangelina Papathoma, Fang Lou, Gabriel Dimitriou, Gerhard Pichler, Giovanni Vento, Gitte Holst Hahn, Gunnar Naulaers, Guoqiang Cheng, Hans Fuchs, Hilal Ozkan, Isabel De Las Cuevas, Iwona Sadowska-Krawczenko, Jakub Tkaczyk, Jan Sirc, Jinhua Zhang, Jonathan Mintzer, Julie De Buyst, Karen McCall, Klaudiusz Bober, Kosmas Sarafidis, Lars Bender, Laura Serrano Lopez, Lina Chalak, Ling Yang, Luc Cornette, Luis Arruza, Mariana Baserga, Martin Stocker, Massimo Agosti, Merih Cetinkaya, Miguel Alsina, Monica Fumagalli, Olalla Lóepez Suarez, Olalla Otero, Olivier Baud, Pamela Zafra, Peter Agergaard, Pierre Maton, Renaud Viellevoye, Ruth del Rio Florentino, Ryszard Lauterbach, Salvador Piris Borregas, Saudamini Nesargi, Segundo Rite, Shashidhar Rao, Shujuan Zeng, Silvia Pisoni, Simon Hyttel-Sørensen, Siv Fredly, Suna Oguz, Tanja Karen, Tomasz Szczapa, Xiaoyan Gao, Xin Xu, Zhaoqing Yin

**Affiliations:** 1 Copenhagen Trial Unit, Centre for Clinical Intervention Research, The Capital Region, Copenhagen University Hospital - Rigshospitalet, Copenhagen, Denmark; 2 Department of Neuroanaesthesiology, Neuroscience Centre, Copenhagen University Hospital - Rigshospitalet, Copenhagen, Denmark; 3 Department of Neonatology, Juliane Marie Centre, Copenhagen University Hospital - Rigshospitalet, Copenhagen, Denmark; 4 Institute of Regional Health Research, Faculty of Health Sciences, University of Southern Denmark, Odense, Denmark

**Keywords:** Central monitoring, Data quality, Data deviations, Missing data, Clinical trials, Mahalanobis distance

## Abstract

**Background:**

Data monitoring of clinical trials is a tool aimed at reducing the risks of random errors (e.g. clerical errors) and systematic errors, which include misinterpretation, misunderstandings, and fabrication. Traditional ‘good clinical practice data monitoring’ with on-site monitors increases trial costs and is time consuming for the local investigators. This paper aims to outline our approach of time-effective central data monitoring for the SafeBoosC-III multicentre randomised clinical trial and present the results from the first three central data monitoring meetings.

**Methods:**

The present approach to central data monitoring was implemented for the SafeBoosC-III trial, a large, pragmatic, multicentre, randomised clinical trial evaluating the benefits and harms of treatment based on cerebral oxygenation monitoring in preterm infants during the first days of life versus monitoring and treatment as usual. We aimed to optimise completeness and quality and to minimise deviations, thereby limiting random and systematic errors. We designed an automated report which was blinded to group allocation, to ease the work of data monitoring. The central data monitoring group first reviewed the data using summary plots only, and thereafter included the results of the multivariate Mahalanobis distance of each centre from the common mean. The decisions of the group were manually added to the reports for dissemination, information, correcting errors, preventing furture errors and documentation.

**Results:**

The first three central monitoring meetings identified 156 entries of interest, decided upon contacting the local investigators for 146 of these, which resulted in correction of 53 entries. Multiple systematic errors and protocol violations were identified, one of these included 103/818 randomised participants. Accordingly, the electronic participant record form (ePRF) was improved to reduce ambiguity.

**Discussion:**

We present a methodology for central data monitoring to optimise quality control and quality development. The initial results included identification of random errors in data entries leading to correction of the ePRF, systematic protocol violations, and potential protocol adherence issues. Central data monitoring may optimise concurrent data completeness and may help timely detection of data deviations due to misunderstandings or fabricated data.

**Supplementary Information:**

The online version contains supplementary material available at 10.1186/s12874-021-01344-4.

## Introduction

‘Good clinical practice data monitoring’ of clinical trials is a tool to ensure high quality and accuracy of the data, and adherence to the trial protocol [1, 2]. Quality and accuracy of the data is threatened by random and systematic errors. Random errors include clerical errors and missing data (when missing at random), and primarily reduce statistical power [3, 4]. Systematic errors, however, may create bias and skew the results [3, 4]. The primary causes of systematic errors are misinterpretation, misunderstandings, and fabrication of data. Hence, these should therefore be the primary focus of data monitoring [3, 5]. Data monitoring with on-site monitors increases trial costs and is time consuming for the local investigators [6–8]. Moreover, during the present COVID-19 pandemic, on-site monitoring has been complicated due to health risks and the different lockdown restrictions [9]. On-site monitoring also has the disadvantage of focusing on data by a case-by-case review, and thereby primarily addressing random errors [10, 11].

In most clinical trials, the local investigators are solely responsible for ensuring quality and accuracy of the data and adherence to the protocol throughout the trial – as checked by on-site monitors [12]. The digital revolution has paved the way for the possibility of *central data monitoring* which can give the coordinating investigator a role in ensuring data quality. Central data monitoring may be conducted in many ways, and should optimally be carried out by a *central data monitoring group* comprising different competences [2, 11, 13]. This group should not assess safety or interventional effects, as this is a task for the Data Monitoring and Safety Committee (DMSC) [14]. This allows the central data monitoring group to remain blinded to group allocation throughout the lifetime of the trial and focus on identifying missing and ‘odd’ data/data patterns, thereby helping to ensure high quality and accuracy of the data on a running basis to replace the work of ‘data cleaning’ operations at the end of the trial. Hence, the central data monitoring group will identify deviations from the protocol and allow timely corrections and improvements of the electronic participant record form (ePRF).

This study aims to outline our approach on the implementation of time-effective *central data monitoring* for the SafeBoosC-III randomised clinical trial to optimise quality control and quality development [15], and present the initial results from the first three central data monitoring meetings.

## Methods

The present approach to central data monitoring was implemented for the SafeBoosC-III trial, a large, pragmatic, multicentre, randomised clinical trial evaluating the benefits and harms of treatment based on cerebral oxygenation monitoring in preterm infants during the first days of life [15]. A total of 1600 extremely preterm infants will be randomised to an experimental versus a control group. In the experimental group, the infants will, as an addition to the usual routine monitoring, undergo cerebral oxygenation monitoring, using near-infrared spectroscopy (NIRS), during the first 3 days of life. If the cerebral oxygenation drops below a predefined hypoxic threshold, the clinician should evaluate the clinical status of the infant and decide on how to intervene, based on a predefined and published physiology-based treatment guideline [15, 16]. Infants randomised to the control group will receive monitoring and treatment as usual. Since less than 0.5% of babies are born extremely preterm, a large of number of centres are needed to reach the predefined sample size [17]. Therefore, more than 70 centres from 18 different countries ranging from high to middle income are participating in the SafeBoosC-III trial. The first infant was randomised in June 2018 and up until the 24^th^ of May 2021, 1070 infants have been randomised. The detailed central data monitoring protocol and reports can be found on the SafeBoosC-III website (https://www.rigshospitalet.dk/english/departments/juliane-marie-centre/department-of-neonatology/research/SafeboosC-III/Sider/good-clinical-practice.aspx).

### Central data monitoring outcomes

#### Data completeness

Missing data is an issue in all fields of research, where just one missing value can end up excluding a participant from a study. Although statistical methods have been developed to minimise the influence of missing data by imputation, these, however, will always be inferior to actual data [4]. The reported results from clinical trials are affected by missing data, which reduces statistical power and could potentially also skew the reported results [4]. The causes of missing data are endless, with only some data being readily retrospectively retrievable. Identification and correction of missing data is a tool, more effective if timely done, but cannot be continuous, as correction is a manual process.

#### Data quality

The quality of the data acquired in a clinical trial depends highly on the ability to follow the protocol. Protocol deviations and protocol violations are known risk factors to clinical trials, and more so to large, multicentre trials [8]. Hence, the use of quality indicators is an integral part of the execution of many clinical trials. Quality measures depict the adherence to the protocol and are used in the interpretation of the results. Quality deficiencies can result in exclusion of participants or even whole centres. Quality deficiencies which are not monitored throughout a trial can result in exclusion of large groups of participants or centres during the post-hoc analyses, but if identified during the trial, they can help optimise the protocol and increase the adherence. Prevention is better than cure.

Quality deficiencies in the SafeBoosC-III central data monitoring include, among others, proportion of participants without an early and a late cranial ultrasound scan, proportion of participants late initiation of cerebral oximetry monitoring, and proportion of participants in the control group that underwent unblinded cerebral oximetry monitoring (Table 1). These quality measures were collected and assessed to help judging the validity of the results. These variables are unique for each trial as they have direct relevance to the interventions and primary outcomes used in a trial.

**Table 1:** Defined quality deficiencies for SafeBoosC-III randomised clinical trial

1.	Proportion of participants without an early and a late cranial ultrasound scan (including only participants alive after 35 days of life)
2.	Late initiation of cerebral oximetry monitoring (0 to 6 h from birth)
3.	Proportion of participants where cerebral oximetry was stopped prematurely (including only participants alive after 72 h of life)
4.	Proportion of participants where consent was withdrawn or declined by the parents
5.	Proportion of participants with a severe brain injury but no cranial ultrasound scan
6.	Proportion of participants with post-haemorrhagic ventricular dilatation or cerebral atrophy but no late cranial ultrasound scan
7.	Proportion of participants in the control group that underwent unblinded cerebral oximetry monitoring

#### Data deviations

Data deviations are defined as 1) *suspected outliers,* e.g. due to random errors in data entries; 2) *suspected misunderstandings* leading to systematic errors in data entries; and 3) *suspected fabricated data*.

*Suspected outliers* are only detectable in continuous variables, by either visual presentation or possibly by presenting the range. Suspected outliers can be caused by a clerical error, suboptimal explained definition of a variable in the ePRF, and/or by different units of measurement used among the centres.

*Suspected misunderstandings* can be identified in both categorical and continuous variables and may be identified by unexpected differences in the expected distributions of data. The expectation is either defined by previous studies or in our multicentre setting in comparison among the centres. These may represent misinterpretation of the coding of data in the ePRF, the overall study design, or simple differences in units of measurement. The definition of a variable, e.g. interpretation of symptoms, might vary depending on the investigator.

*Suspected fabricated data* is defined as an unexpected distribution of data, which cannot be explained by any clinical differences nor by any of the above-mentioned deviations. Continuous data could have a different shape or distribution when illustrated, as natural variance is difficult to fabricate [18]. The categorical variables might also show an unexpectedly low or high occurence, compared to both previous literature and comparable centres. Fabricated data is difficult to identify by looking at one variable at a time. However, multivariable statistical models can help identify potential centres with suspected fabricated data, which hereafter should be further investigated for each of the variables and/or participants [11].

### Central data monitoring group

The *central data monitoring group* serves as data reviewers. The sole purpose of this group was evaluation of data completeness, quality, and deviations. Our group consists of the trial manager, one experienced clinician in the field of neonatology, one statistician or data scientist, and two trialists. This diversity ensured the required expertise to interpret the data, and the likely best approach as to when to intervene. Members of the central data monitoring group were not members of the Data and Safety Monitoring Committee (DSMC), as the independence and objectivity of the DSMC could be affected if this were the case [19]. The members of the central data monitoring group are blinded to group allocation, and during the meetings also blinded to which centre the data is originating from by the use of a randomly generated acronym for each centre.

### Central data monitoring reports

The data completeness and data quality reports, generated using R version 4.0.3 (R Core Team, Vienna, Austria) together with Rmarkdown [20], are separate and generated with different frequency. Any changes in the code from the initial reports are noted in a changelog.

#### Data completeness report

**Fig. 1:**
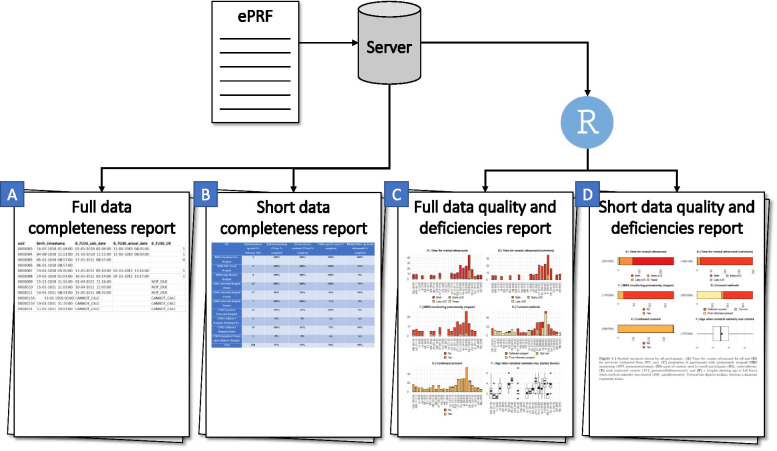
Generation of the central data monitoring reports utilises R and markdown. The full reports, i.e. **A ***full data completeness report* and **C ***full data quality and deficiencies report* are utilised by the data monitoring group, while the short reports, i.e. **B ***short data completeness report* and **D ***short data quality and deficiencies report*, are used for newsletters and benchmarking. ePRF: electronic Participant Report Forms

The *full data completeness report* is used by the trial manager in the central data monitoring group as a tool to detect missing data and getting in contact with the local investigators. The data extract shows, for each data entry module for each participant: a) the date where the data entry module should be completed; b) the actual completion date of the data entry module; and c) whether or not the data entry module has been completed (Fig. 1A).

The *short data completeness report* summarises the full data completeness report at anonymised site level. The report depicts the completion percentage from each centre where the numerator was all participants with complete entries and denominator is all participants. Thus, if one centre has completed 36-week data for 17 participants but a total of 20 follow-up participants should have been completed, the completion proportion of this instrument for this centre is 17/20 = 0.85.

The summarized report is included in the monthly newsletters to the local investigators (https://www.rigshospitalet.dk/english/departments/juliane-marie-centre/department-of-neonatology/research/SafeboosC-III/Sider/good-clinical-practice.aspx; Supplemental file 1) and as benchmarking for the Steering Committee (Fig. 1B).

#### Data quality and deficiencies report

**Fig. 2:**
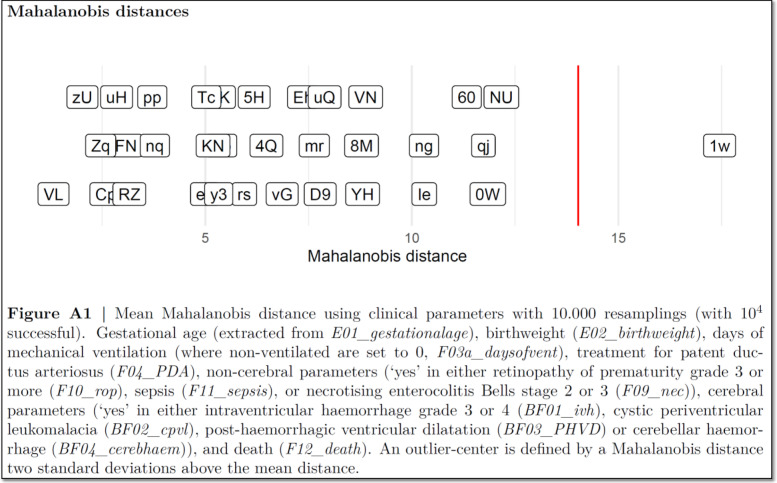
This is an example of how the Mahalanobis distance is presented in the *full data quality and deficiencies reports*. The centres are presented with blinded acronyms, which are used throughout the central data monitoring meetings

The *full data quality and deficiencies report* is used during the data monitoring meetings where the data for each variable are presented, stratified by centres but with the two randomisation groups combined (Fig. 1C). In a multicentre trial, such as SafeBoosC-III, where the variables are predominantly categorical, data quality and deviations are nearly impossible to assess in centres with few entries, and we therefore decided that centres with less than 5 included participants would not be included in the report, and only variables with 5 or more entries of the specific variable should be presented. Misunderstanding or fabricated data may occur for many of the variables, and interpretation of ‘odd’ patterns in the data is difficult without any statistical guidance. Many statistical models have been suggested in order to identify fabricated data, which predominantly monitors continuous variables [18, 21]. We chose Mahalanobis distance (see below) which seemed best suited for multivariable pattern-anomaly detection in our setting [22]. We chose the variables related to the characteristics of the participants, intervention, quality measures, and outcomes, which included 31 variables. Together with the assessment of each variable, Mahalanobis multivariate distance is used to ‘raise a suspicion’ of centre-specific differences. These differences can be due to differences between the centres in patient population, in policies for clinical management, or actual systematic errors. We examined the data twice, once without knowledge of the Mahalanobis distance and once after presentation of the Mahalanobis distance (Fig. 2). Any deviation and the decision of whether an action should be initiated is noted in the *short data quality and deficiencies report*.

**Fig. 3:**
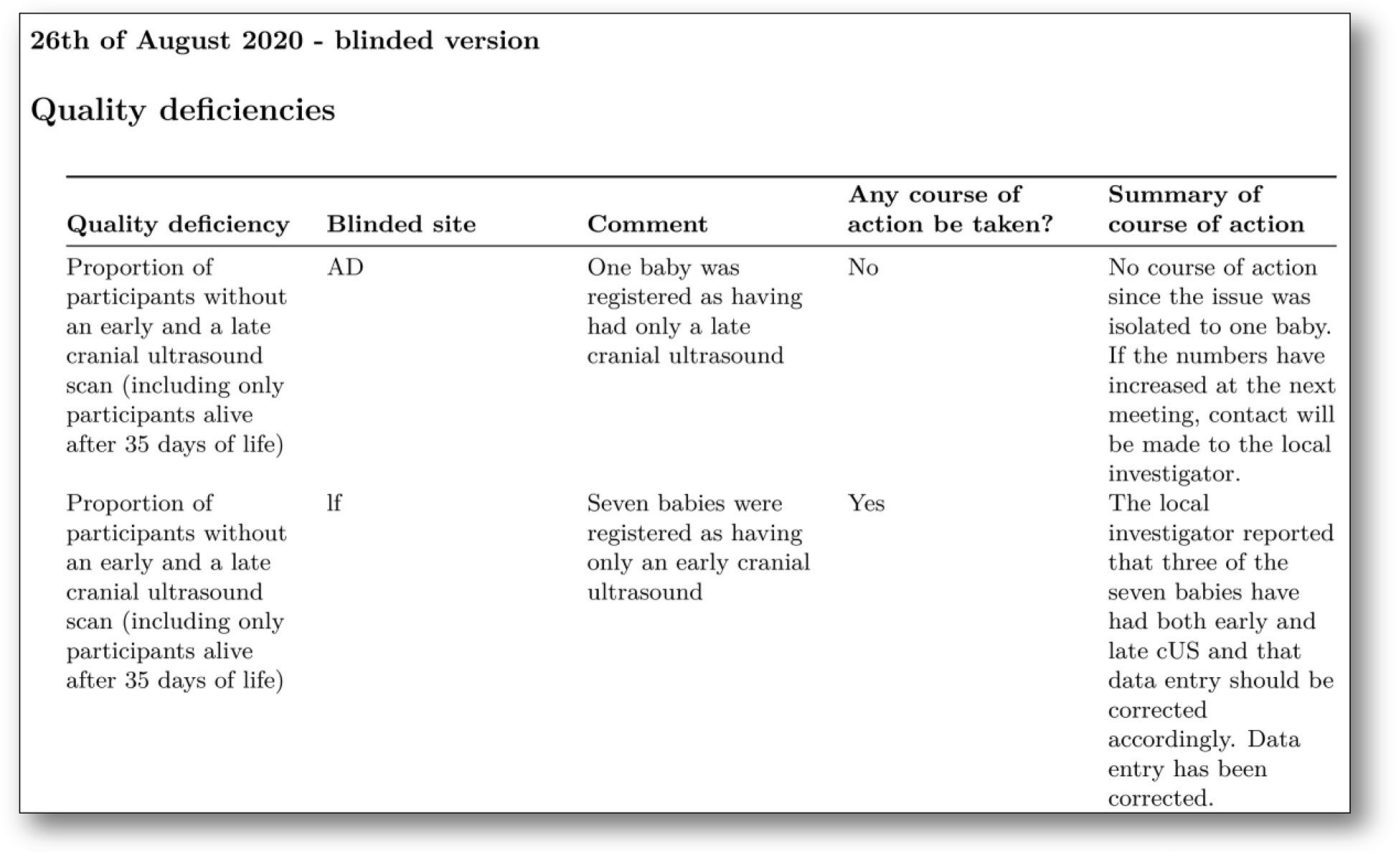
This is a part of the first central data monitoring log which exemplify the flags, and the course of action for two of the flags

The *short data quality and deficiencies report* is divided into two parts: the first with data presentation only, and the second with decisions made by the data monitoring group. The variables monitored are presented without any stratification, i.e. results from all centres and both groups combined. The conclusion from the meetings together with results from the course of action is also included in the report (Fig. 3). The reports are publicly available (https://www.rigshospitalet.dk/english/departments/juliane-marie-centre/department-of-neonatology/research/SafeboosC-III/Sider/good-clinical-practice.aspx; Supplemental file 2) and used for benchmarking purposes, similarly to the short data completeness report (Fig. 1D).

#### Mahalanobis distance

The Mahalanobis distance is a parameter of a statistical model which allows for identification of statistical outliers in a multidimensional space [22]. The distance is a measure (in the unit of standard deviations) of how far a single point is from the multivariate mean in the multidimensional space [22]. The definition of an outlier varies, depending on the purpose of the analysis. Some authors define an absolute distance (e.g. 2 standard deviations (SD)) before a measurement is an outlier, while others work with cut-offs depending on the sample size [18, 22]. Here, we used two standard deviations. Sites with data from less than 5 participants at the time of analysis were excluded [18, 23]. For every site, participants were randomly re-sampled (Monte-Carlo simulation) and a new mean for continuous values and a new proportion for binary features were calculated. Every centre is then compared using a multivariable Mahalanobis analysis [22]. This re-sampling and calculation were repeated 10,000 times to provide robust estimates of the sampling error, for judging whether a centre is outside the defined limit of 2 standard deviations.

### Central data monitoring activities

The responsibility of the central data monitoring group is split into data completeness monitoring, and data quality and deviation monitoring. Data completeness monitoring was initiated when 100 participants had been randomised, while data quality and deviation monitoring was initiated after 400 of the participants were randomised and is performed less frequently than the data completeness monitoring.

#### Data completeness

Data completeness monitoring is carried out monthly which is frequent enough for the local investigators to remember the participants if any values are not easily extractable from the charts, and seldom enough, so local investigators have time to complete the entries. Stable and regular assessment of completeness ensures that the local investigators can meet the deadlines for each entry. Our approach is a zero tolerance of missing data, being the ethical approach to clinical trials, where any missing data is sought completed by contacting local investigators.

#### Data quality and deviations

Data quality and deviations monitoring is carried out on a trimonthly basis, providing time to implement changes between each monitoring meeting. These monitoring meetings can result in corrections in multiple data entries, structural changes to the database, or potential changes to standard operating procedures or to the protocol itself. The changes to the database can include new variable explanations, definitions, or changing allowed intervals for continuous variables. The assessments of data quality and deviation are more subjective than data completeness monitoring, which is why the central data monitoring group should discuss the findings. The purpose of these meetings is to identify anomalies and interpret if they were due to centre or population specific differences or if suspected outliers, misunderstanding, or fabricated data are the cause. Furthermore, the course of action from each of these anomalies is decided at these meetings. The meetings were set to last approximately 2 h and included all members of the *central data monitoring group*.

## Results

**Fig. 4:**
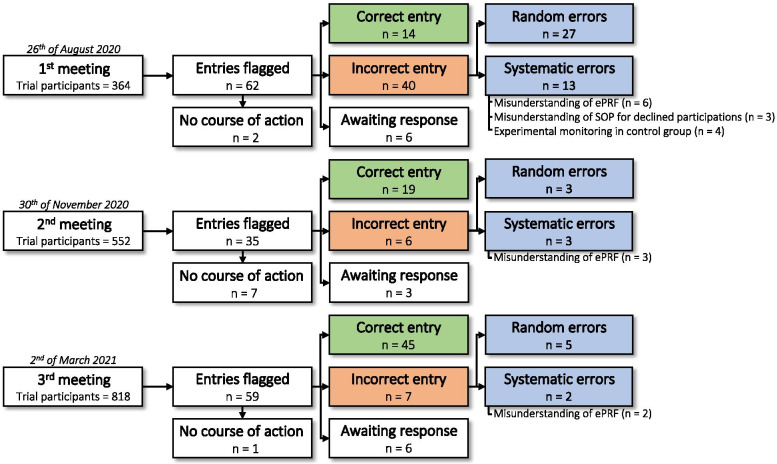
These diagrams show the results from the first three meeting. The first column shows how many participants were included, and the second row show the number of entries which were flagged, and furthermore, in how many where an action was not deemed necessary. The investigators contacted received information about the entries which were flagged and an explanation of standard operating procedure. The last column present if an entry was correct or incorrect after response from the local investigator. ePRF: electronic participation report form; SOP: standard operating procedure

After launch of the SafeBoosC-III trial, the central data monitoring group was created (consisting of MHO, MLH, JCJ, GG, and CG), and a protocol for central data monitoring was developed (available at https://www.rigshospitalet.dk/english/departments/juliane-marie-centre/department-of-neonatology/research/SafeboosC-III/Sider/good-clinical-practice.aspx). The *full data quality and deficiencies report* was used during the central data monitoring meetings. In the first three meetings, the central data monitoring group identified 156 data entries of potential interest and decided upon contacting the local investigators for 146 (94%) of these. These consultations resulted in correction of 53 (36%) entries (Fig. 4).

After the first meeting, three centres were identified with suspected misunderstandings leading to systematic protocol violations with NIRS monitoring of the participants in the control group, which is only allowed in the experimental group. This suspicion was confirmed by the local investigators, and they ceased this practice. We, furthermore, identified many centres with incorrect entries of a ‘consent method’, which was not approved for the specific centre. This led to amendments of the ePRF, allowing only data entry of a consent method that was approved by the local ethics committee for the given centre. In one centre, the “proportion of participants without an early and a late cranial ultrasound scan” was especially high (Table 1; #1). The trial manager contacted the local investigator to ask whether these data entries were correct. This resulted in a review of the clinical records by the local investigator who identified three of the eight babies having incorrect data entry in the ePRF, since the three babies had undergone both an early and a late scan. The ePRF was accordingly amended to prevent such errors. Figure 3 depicts how such course of action is registered in the monitoring log.

The second meeting identified that the proportion of participants where management was adjusted due to cerebral hypoxia was lower in the centres with only a few included participants. This possible systematic error resulted in a reminder for all investigators, to follow the algorithms investigated in the trial.

During the third meeting we identified multiple survivors with low weight at follow-up and decided to create a weight curve of the participants. This identified that the follow-up of 103/818 participants was registered after the predefined follow-up date. The follow-up date in SafeBoosC-III is defined as an assessment at the time the participants reach 36 weeks postmenstrual age (PMA). This systematic error was present across multiple centres and can potentially skew the results of the primary outcome. This issue was addressed by the Steering Committee of the trial, which decided the following:

*“As the majority of cases have a follow-up date that is only a few days after 36*+*0 weeks PMA and the potential effect of an outcome assessment bias is small since very few babies die around 36*+*0 weeks PMA, we decide not to do any further. However, OpenClinica* [the ePRF] *will be revised so that it is no longer possible to enter a follow-up date later than 36*+*0 weeks PMA”*

This change allowed the investigators to enter data at a date later than the follow-up date but were forced to enter the date the data referred to. All the findings are summarised in the *short data quality and deficiencies report* and publicly available (https://www.rigshospitalet.dk/english/departments/juliane-marie-centre/department-of-neonatology/research/SafeboosC-III/Sider/good-clinical-practice.aspx; Supplemental file 2).

## Discussion

This pilot study outlines a time-effective methodology of *central data monitoring* and presents some initial results, which included identification of random errors in data entries leading to correction of the ePRF, systematic protocol violations, and potential protocol adherence issues. This being done throughout the trial, as preventive measures may increase the validity of the final results.

The addition of a *central data monitoring group*, which frequently evaluate data from the clinical trial, may be an effective part of large clinical trials to improve data control and quality development. The creation of such a group allows for time-effective and low-cost data optimisation. The preliminary results confirm that our approach to central data monitoring may minimise both random errors and systematic errors, thereby increasing the validity of our results [2, 3].

Our methodology combined both expertise and statistical monitoring. By combining manual investigation with statistical monitoring, we identified both random and systematic errors [11, 18, 24]. This is the first step towards a warranted and necessary standardised approach to central data monitoring [25].

Even with optimal trial design and planning to minimise errors, unforeseen challenges can still occur. Countries, hospitals, departments, and investigators differ, therefore thorough explanation of symptoms and diagnoses should be readily available in the ePRF. The room for misunderstandings is immense, such as different decimal marker, 12/24-h clocks, and month at the beginning of a date string. The risk of such misunderstandings increases with the number of centres involved. Thus, frequent central evaluation of data may show its worth.

DSMCs are often responsible for all data monitoring during a trial, but the primary responsibility is to represent the interests of the participants, in particular their safety, i.e. to judge whether a trial should continue, stop, or be modified in the light of accumulating adverse events and/or interim analysis of the effect of the intervention under test [26, 27]. The implementation of these important activities and actions in the SafeBoosC-III trial is not reported here. The independence of a DSMC is necessary to ensure minimal conflict of interest in their safety assessment, which is its primary purpose [28, 29]. In contrast, the central data monitoring group represent the interests of the trial. Importantly, this work can be conducted blinding to randomisation groups, and thus without compromising ignorance about the balance of evidence for or against the intervention that is tested, and therefore be implemented as an integrated element of central trial management.

The additional bonus of a central data monitoring group is that the obtained trial data are already cleaned and corrected as soon as the inclusion and follow-up ends, and discussions about centre-specific differences regarding data peculiarities have already been carried out. Central data monitoring will allow for almost immediate statistical analysis of the trial results and facilitate faster publication.

Although we found the model of central data monitoring effective, we cannot claim that it is superior to other models. Our intent by this publication is to give sufficient detail to allow randomised comparative studies in the future. Reports of such are missing in the literature.

In conclusion, central data monitoring is made possible by web-based, real-time data entry, where all the data are stored centrally. Central data monitoring may optimise concurrent data completeness and quality and help timely detection of data deviations due to misunderstandings or fabricated data. Central data monitoring is particularly relevant during the restrictions due to the COVID-19 pandemic and should be considered for clinical trials that are currently being executed.

## Supplementary Information

**Additional file 1.**

**Additional file 2.**

## Data Availability

The datasets will be made available after reasonable request to the coordinating investigator of SafeBoosC-III, while the scripts for the reports will be made available after reasonable request to the corresponding author.
