# Supplementary material for: Central data monitoring in the multicentre randomised SafeBoosC-III trial – a pragmatic approach
Source: BMC Med Res Methodol. 2021 Jul 31;21:160. doi: 10.1186/s12874-021-01344-4 (PMC8325420; doi:10.1186/s12874-021-01344-4)
Supplement: Supplementary file 1 — Additional file 1. [file 12874_2021_1344_MOESM1_ESM.pdf]

SafeBoosC III Data completion report

18-March 2021

The data shown below is based on all registered data entries in OpenClinica on SafeBoosC III trial participants, up until 18th of March 2021. The data has been analysed and reported according to the SafeBoosC III Central monitoring plan (see www.safeboosc.eu under "Good Clinical Practice").

Direct link to the Central monitoring plan: https://www.rigshospitalet.dk/english/departments/juliane-marie-centre/department-of-neonatology/research/SafeboosC-III/Documents/central-monitoring-plan.pdf

Completion of data entries

Below you will find an overview of data entries across all centres.

Note that completion calculations are based on babies that have reached at least 10 days of life for end-of-monitoring completion, and 40 weeks of postmenstrual age (PMA) for Follow-up and Blinded follow-up completion. n/a = not applicable, meaning that no babies at the specific site has reached 10 days of life or 40 weeks of PMA when applicable.

Site	Randomisations up until 18-March 2021	End-of-monitoring (72 hrs) % completed	Serious adverse reactions (72 hrs) % completed	Follow-up (36 weeks) % completed	Blinded follow-up (brain ultrasound) % completed
AT01 Univ. Hospital Graz	12	100%	100%	100%	80%
BE01 Univ. Hospital Leuven	24	100%	100%	85%	92%
BE02 AZ St. Jain Univ. Hosp. Bruges	7	100%	0%	100%	100%

Site	Randomisations up until 18-March 2021	End-of-monitoring (72 hrs) % completed	Serious adverse reactions (72 hrs) % completed	Follow-up (36 weeks) % completed	Blinded follow-up (brain ultrasound) % completed
BE03 Charleroi Univ. Hospital	5	100%	100%	80%	80%
BE04 CHU Tivoli Hospital	9	100%	100%	100%	75%
BE06 Liege Rocourt Hospital	10	70%	40%	50%	0%
CH01 University Hospital Zürich	33	100%	100%	100%	71%
CH03 University Hospital Lucern	31	89%	85%	90%	95%
CH04 University Hospital Geneva	8	88%	67%	100%	0%
CH05 Lausanne University Hospital	13	100%	20%	60%	0%
CN01 Children's Hospital, Zheijang Univ.	2	0%	0%	n/a	n/a
CN02 Children's Hospital, Fudan	35	97%	81%	86%	86%
CN04 Guangzhou Women and Children's Hospital	2	0%	0%	n/a	n/a
Total	891	97%	92%	97%	91%

Site	Randomisations up until 18-March 2021	End-of-monitoring (72 hrs) % completed	Serious adverse reactions (72 hrs) % completed	Follow-up (36 weeks) % completed	Blinded follow-up (brain ultrasound) % completed
CN05 Longgang Distr. Centr. Hosp. Shenzen	7	100%	100%	100%	100%
CN06 Xiamen Children's Hospital	2	100%	100%	0%	100%
CN07 The People's Hospital of Dehong	3	100%	100%	100%	100%
CN08 Maternal and Child Health Hosp. Quangxi	5	100%	100%	100%	100%
CZ01 The Institute for the Care of Mother and Child	46	100%	85%	100%	97%
CZ02 Motol Univ. Hospital	1	100%	n/a	n/a	n/a
DE01 University Hospital Freiburg	6	100%	100%	100%	100%
DK01 Rigshospitalet	78	100%	100%	100%	100%
DK04 Aalborg University Hospital	8	100%	100%	100%	80%
Total	891	97%	92%	97%	91%

Site	Randomisations up until 18-March 2021	End-of-monitoring (72 hrs) % completed	Serious adverse reactions (72 hrs) % completed	Follow-up (36 weeks) % completed	Blinded follow-up (brain ultrasound) % completed
DK18 Aarhus University Hospital	10	100%	100%	100%	71%
DK30 Odense University Hospital	9	50%	0%	50%	0%
ES01 La Paz University Hospital	60	100%	100%	100%	100%
ES02 Hospital Clinic de Barcelona (Maternitat)	37	100%	100%	100%	100%
ES03 University Hospital 12 de Octubre	38	100%	100%	100%	100%
ES05 Hospital de Sant Joan de Deu	24	100%	100%	100%	100%
ES06 H. U. Puerta del Mar	17	100%	100%	100%	100%
ES08 Hospital Clinico San Carlos	25	100%	100%	100%	100%
ES09 H. U. Marques de Valdecilla	14	100%	100%	100%	100%
ES10 Virgen de las Nieves	6	100%	100%	100%	100%
Total	891	97%	92%	97%	91%

Site	Randomisations up until 18-March 2021	End-of-monitoring (72 hrs) % completed	Serious adverse reactions (72 hrs) % completed	Follow-up (36 weeks) % completed	Blinded follow-up (brain ultrasound) % completed
**ES11 H. Univ. Juan XXIII Tarragona**	**11**	**100%**	**100%**	**100%**	**100%**
**ES13 Hospital de Cruces**	**1**	**n/a**	**n/a**	**n/a**	**n/a**
**GR01 Alexandra Hospital, Athens**	**13**	**100%**	**100%**	**100%**	**100%**
**GR02 Ippokrateion Hospital of Thessalonikki**	**25**	**100%**	**100%**	**100%**	**100%**
**GR03 Univ of Patras, General Hospital**	**8**	**100%**	**100%**	**100%**	**100%**
**GR04 Univ Hospital of Heraklion**	**8**	**100%**	**100%**	**100%**	**100%**
**IE01 Univ. College Cork**	**3**	**50%**	**50%**	**n/a**	**n/a**
**IN01 St Johns Medical College Hospital, Bangalore**	**3**	**100%**	**100%**	**100%**	**100%**
**IT01 Presidio Ospedale Sant'Anna, Turin**	**3**	**100%**	**100%**	**100%**	**100%**
**Total**	**891**	**97%**	**92%**	**97%**	**91%**

Site	Randomisations up until 18-March 2021	End-of-monitoring (72 hrs) % completed	Serious adverse reactions (72 hrs) % completed	Follow-up (36 weeks) % completed	Blinded follow-up (brain ultrasound) % completed
IT07 Fondazione IRCCS Milano	31	100%	100%	100%	100%
IT08 Ospedale del Ponte, Varese	4	100%	0%	n/a	n/a
IT09 Fondaz. Policlinico Univ. A Gemelli	4	100%	0%	n/a	n/a
PL01 Medical Center UJASTEK Krakow	24	100%	100%	100%	100%
PL03 Specialist Hospital No. 2 Bytow	3	100%	n/a	100%	100%
PL04 Poznan Univ. of Medical Sciences	23	100%	100%	100%	74%
PL07 Warsaw Univ. Medical Sciences	5	75%	50%	0%	0%
PL08 Szpital Uniwersytecki, Krakow	4	100%	100%	n/a	n/a
TR01 Gazi University Hospital	10	100%	100%	100%	100%
Total	891	97%	92%	97%	91%

Site	Randomisations up until 18-March 2021	End-of-monitoring (72 hrs) % completed	Serious adverse reactions (72 hrs) % completed	Follow-up (36 weeks) % completed	Blinded follow-up (brain ultrasound) % completed
**TR02 Marmara University Hospital**	**20**	**95%**	**100%**	**100%**	**100%**
**TR03 Uludag University Hospital**	**19**	**100%**	**100%**	**100%**	**100%**
**TR04 Kanuni Sultan Hospital**	**11**	**100%**	**100%**	**100%**	**100%**
**TR05 Bilkent, Ankara City Hospital**	**30**	**93%**	**96%**	**95%**	**64%**
**TR06 Basaksehir City Hospital**	**1**	**100%**	**n/a**	**n/a**	**n/a**
**UK08 Royal Hospital for Children, Glasgow**	**3**	**100%**	**100%**	**n/a**	**n/a**
**UK09 NHS Lanarkshire Hospital**	**1**	**n/a**	**n/a**	**n/a**	**n/a**
**US02 Loma Linda Univresity Hospital**	**21**	**100%**	**100%**	**100%**	**88%**
**US03 University of Utah, Div. Neonatology**	**12**	**100%**	**100%**	**100%**	**100%**
**US04 UT Southwestern Medical Center**	**3**	**33%**	**33%**	**n/a**	**n/a**
**Total**	**891**	**97%**	**92%**	**97%**	**91%**

Note that completion calculations are based on babies that have reached at least 10 days of life for end-of-monitoring completion, and 40 weeks of postmenstrual age (PMA) for Follow-up and Blinded follow-up completion. n/a = not applicable, meaning that no babies at the specific site has reached 10 days of life or 40 weeks of PMA when applicable.

Investigators who do not have 100% completion of data entries across all three modules will be contacted by e-mail and asked to enter the missing data into OpenClinica.

Copenhagen Trial Unit, Gorm Greisen and Mathias Lühr Hansen
