# Supplementary material for: Central data monitoring in the multicentre randomised SafeBoosC-III trial – a pragmatic approach
Source: BMC Med Res Methodol. 2021 Jul 31;21:160. doi: 10.1186/s12874-021-01344-4 (PMC8325420; doi:10.1186/s12874-021-01344-4)
Supplement: Supplementary file 2 — Additional file 2. [file 12874_2021_1344_MOESM2_ESM.pdf]

SafeBoosC-III - central monitoring

Olsen MH, Hansen ML, Safi S, Jakobsen JC, Greisen G & Gluud C

25 September, 2020

Contents

Introduction . . . 2
Methods and material . . . 2
Quality measures . . . 3
Randomisation . . . 4
End of monitoring . . . 5
Follow-up (36 weeks postmenstrual age or discharge to home) . . . 7
Blinded follow-up (36 weeks post menstrual age or discharge to home) . . . 9

Central monitoring log **10**
Quality deficiencies . . . 10
Noteworthy data deviations . . . 15
Statistical outlier identification by Mahalanobis distance . . . 16
Changes to the central monitoring report setup for November meeting . . . 17
NOTA . . . 18

Introduction

The data report is generated automatically from data entered into the electronic case report forms (eCRF) related to the SafeBoosC-III trial. Missing data are attended to in another report. The data report is used every third month to monitor quality deficiencies, and noteworthy data deviations. Furthermore, an exploratory Mahalanobis distance will be used to detect potentiel outlier-centres. The data will be examinated by the trial manager and coordinating investigator of SafeBoosC-III and collaborators from Copenhagen Trial Unit (CTU). Any identified quality deficiencies, noteworthy data deviations and outlying centres will be noted in the central monitoring log and discussed with the local investigator. Results from the monitoring will be logged in the central monitoring log.

The protocol for the central monitoring plan and this report will be uploaded to the SafeBoosC-III website (www.safeboosc.eu).

Methods and material

The data report will be generated automatically after extraction of data from the eCRF every three months *(data extracted 17th of august 2020)*. Data from centres with less than five included participants will be excluded since systematic errors and flaws will not be identifiable for small sample sizes.

Participants included in SafeBoosC-III are depicted in boxplots for continous data and stacked barcharts for categorical. Missing data are removed from the output, since these are handled in a separate monthly report. Boxplots are presented with median line and with the interquartile range as hinges. Mean is presented as a diamond.

The data report is generated using R version 4.0.0 (R Core Team, Vienna, Austria) together with Rmarkdown [Allaire et al., 2020]. The code might change during the course of the study, but any changes of data presented and analyses will be approved by the monitoring committee. These changes to the code will be recorded in the central monitoring log.

Quality measures

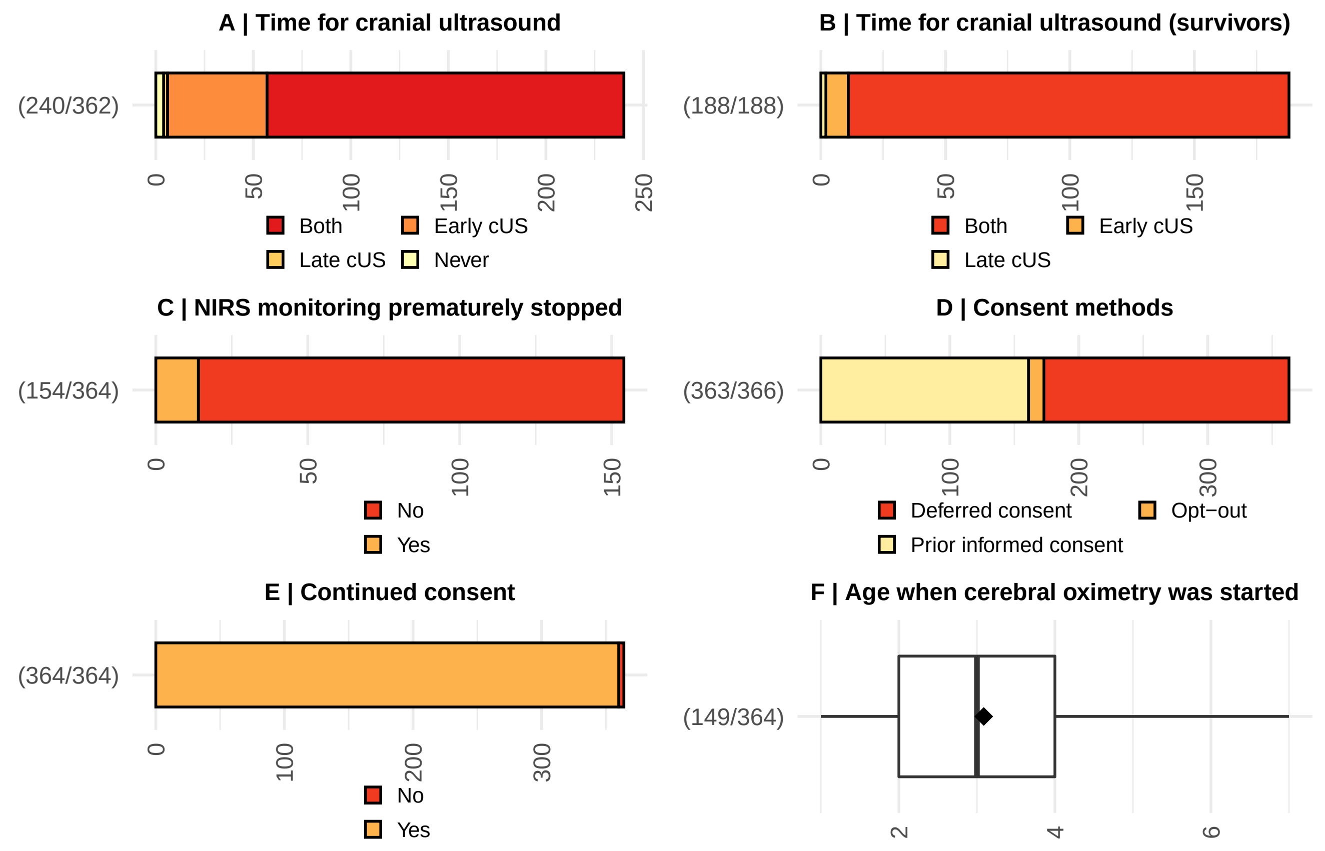

Figure 1 | Stacked barcharts shown for all participants. **(A)** Time for cranial ultrasound for all and **(B)** for survivors (extracted from *F07_cus*); **(C)** proportion of participants with prematurely stopped NIRS monitoring (*E07_prematurenirsstop*); **(D)** types of consent used to enroll participants (*R04_consentform*); **(E)** with continued consent (*E12_parentswithdrawconsent*); and **(F)** a boxplot showing age in full hours when cerebral oximetry was started (*E06_ageinhoursnirs*). Vertical line depicts median, whereas a diamond represents mean.

Randomisation

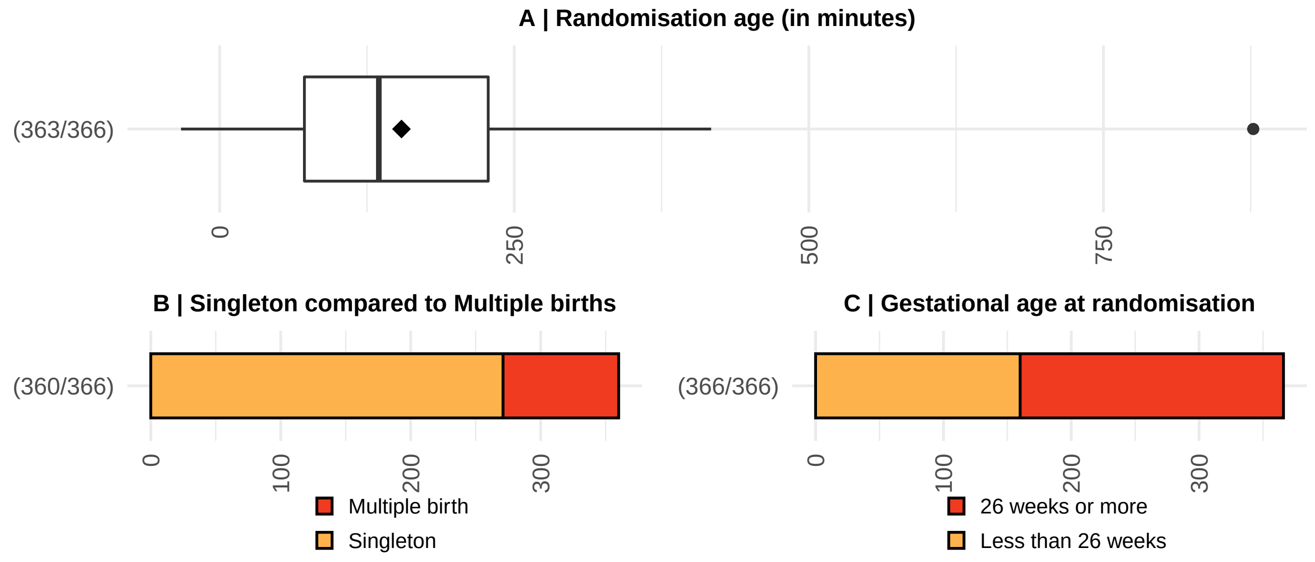

Figure 2 | **(A)** Age of participants at the time of randomisation in minutes presented using a boxplot. Vertical line depicts median, whereas a diamond represents mean. Stacked barcharts from the 'randomisation' module. **(B)** Proportion of singleton compared to multiple births (extracted from *R02a_singlemulti*); and **(C)** gestational age of participants at randomisation (*R07_galessthan26wks*).

End of monitoring

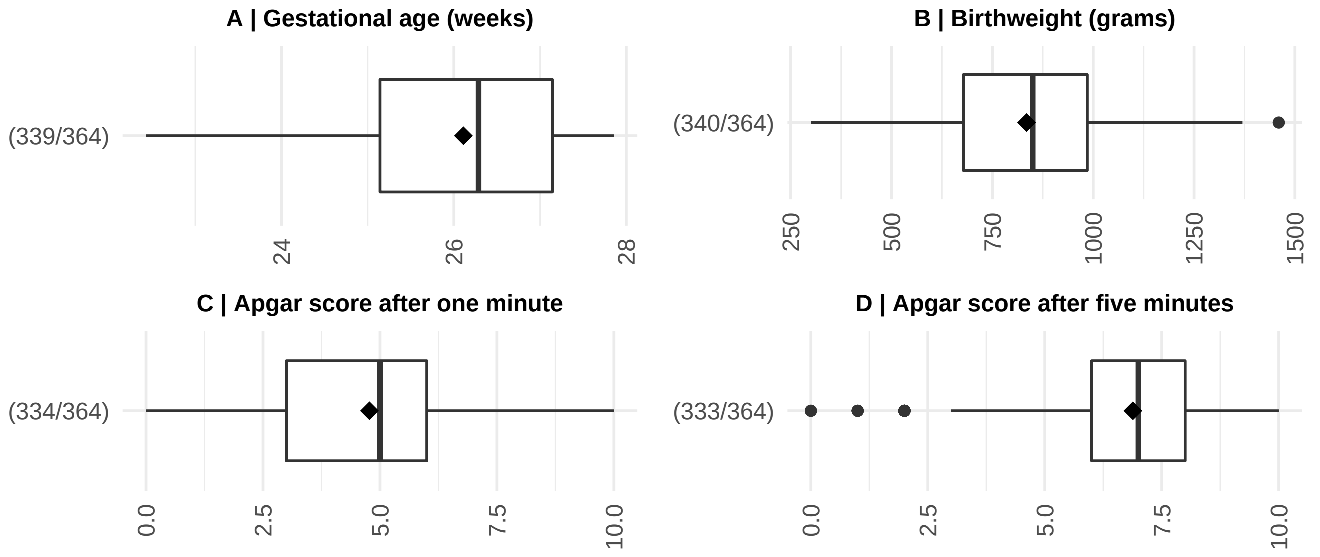

Figure 3 | Boxplots from the 'end of monitoring' module, shown for all participants. **(A)** Gestational age of participants in gestational weeks (extracted from *E01_gestationalage*); **(B)** birthweight in grams of participants (*E02_birthweight*); **(C)** Apgar score for participants one minute after birth (*E03_apgar1min*); and **(D)** Apgar score for participants five minutes after birth (*E04_apgar5min*).

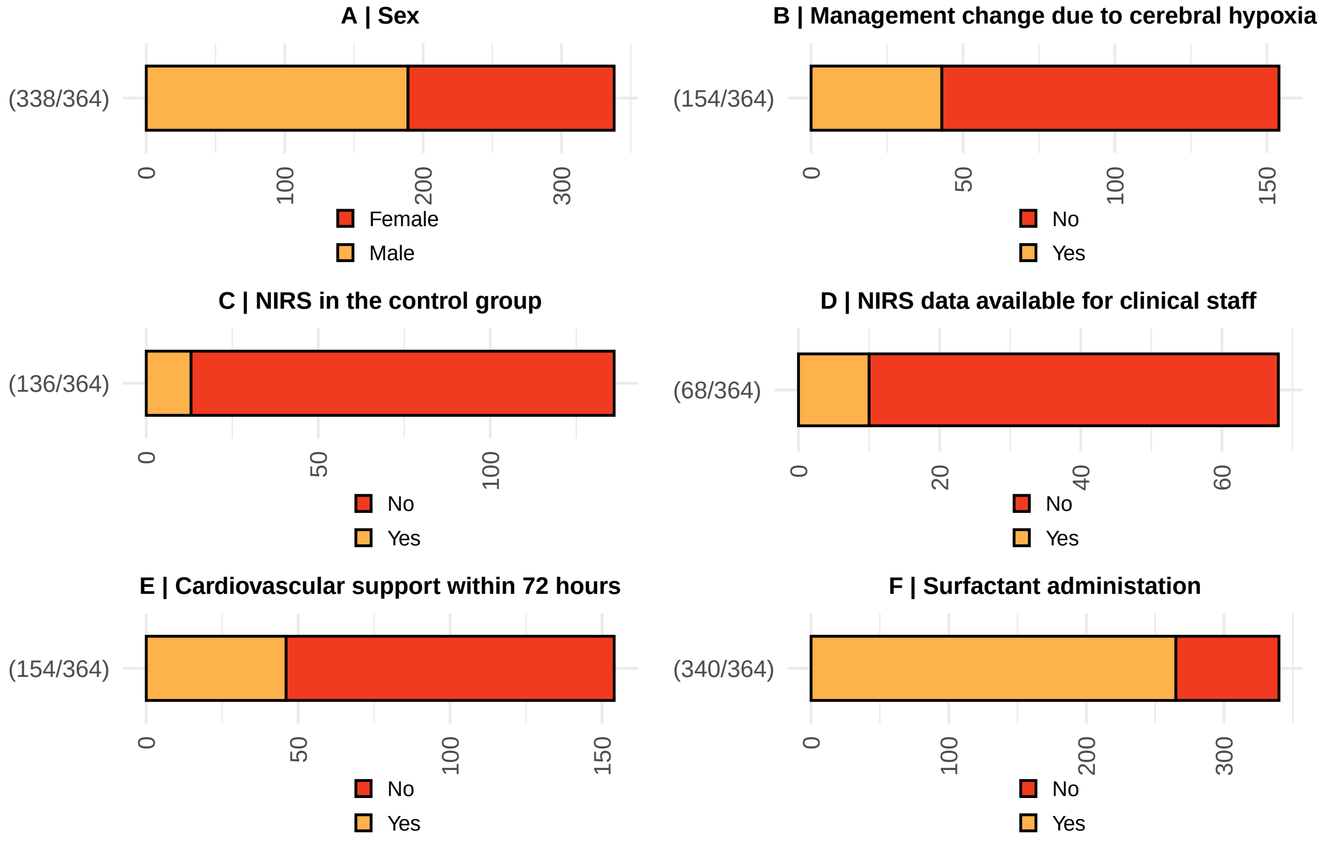

Figure 4 | Stacked barcharts from the 'end of monitoring' module, shown for all participants. **(A)** Sex of particiapants (extracted from *E05_sex*); **(B)** Proportion of participants with changed treatment due to cerebral hypoxia (*E08_changeoftreatmenthypoxia*); **(C)** with NIRS despite being in the control group (*E11_nirsincontrol*); **(D)** where NIRS was available for the clinical staff (*E11a_nirsdata*); **(E)** who recieved cardiovascular support during the first 72 hours after birth (*E09_cardiovascsupp*); and **(F)** who recieved surfactant administration (*E13_surfterap*).

Follow-up (36 weeks postmenstrual age or discharge to home)

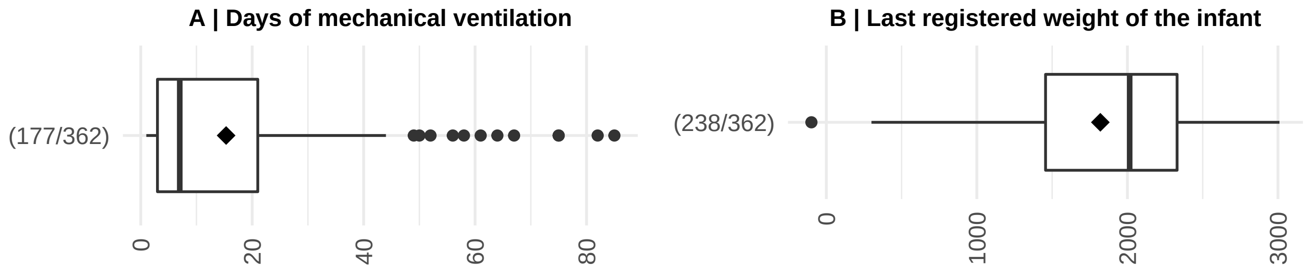

Figure 5 | Boxplots from the 'follow-up' module, shown for all participants. **(A)** Days of mechnical ventilation (extracted from *F03a_daysofvent*); and **(B)** weight at follow-up (*F05_weightatfollowup*).

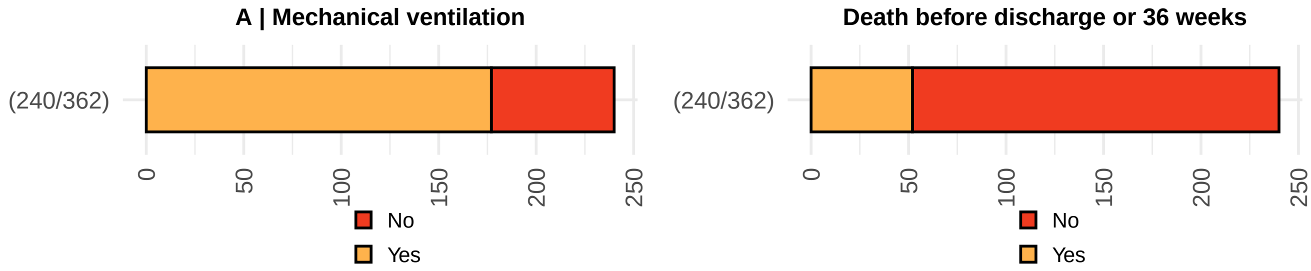

Figure 6 | Stacked barcharts from the 'follow-up' mobule, shown for all participants. **(A)** Proportion of participants on mechanical ventilation during admission (extracted from *F03_mechanicvent*); and **(B)** who died before discharge or before 36 weeks (*F12_death*).

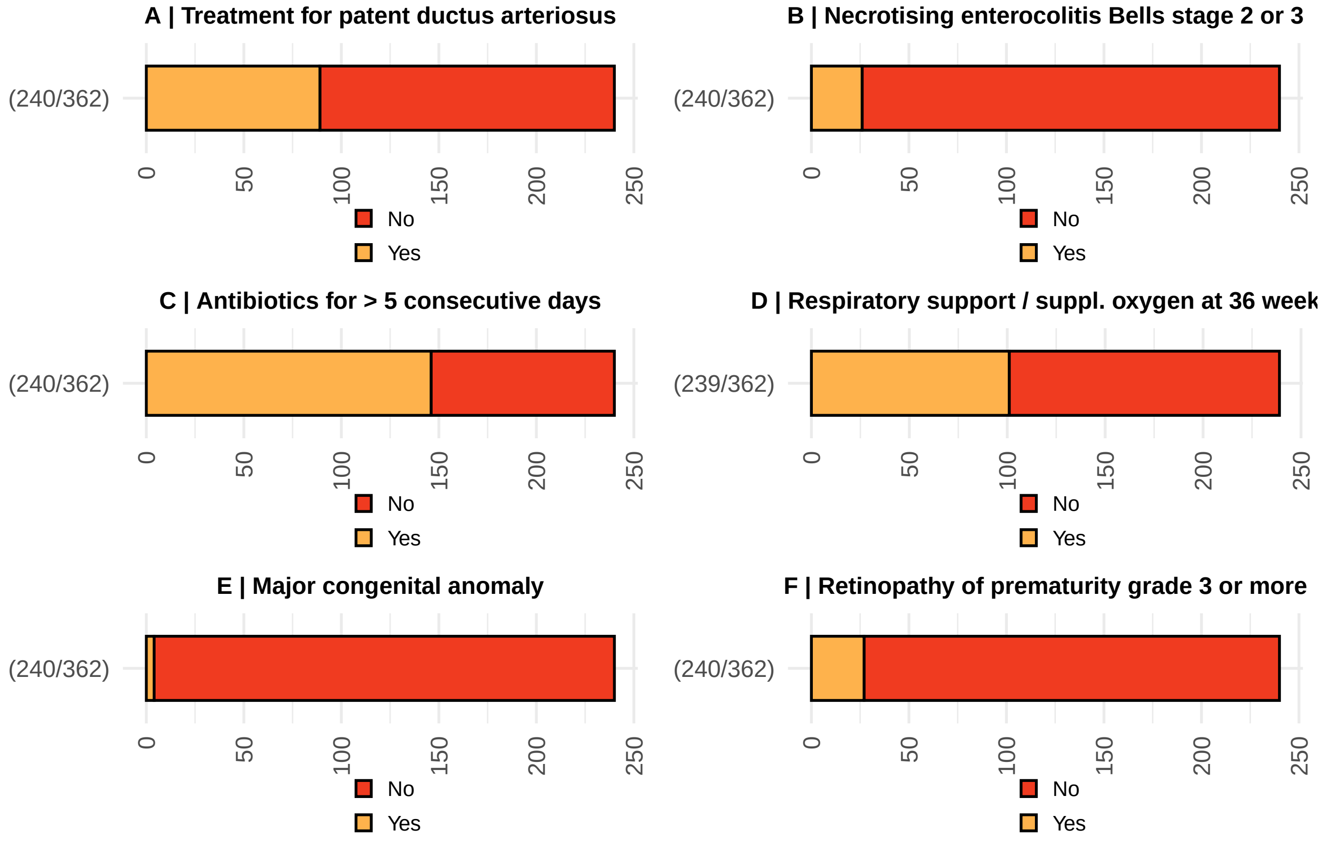

Figure 7 | Stacked barcharts from the 'follow-up' module, shown for all participants. **(A)** Proportion of participants who recieved treatment for patent ductus arteriosus (extracted from *F04_PDA*); **(B)** with necrotising enterocolitis Bells stage 2 or 3 (*F09_nec*); **(C)** who recieved antibiotics for more than five consecutive days (*F11_sepsis*); **(D)** proportion of participants who recieved respiratory support or suplemental oxygen at 36 weeks (*F08_respsupp36wk*); **(E)** with major congenital anomaly (*F02_major_congenitalanomaly*); and **(F)** with retinopathy of prematurity grade 3 or more (*F10_rop*).

Blinded follow-up (36 weeks post menstrual age or discharge to home)

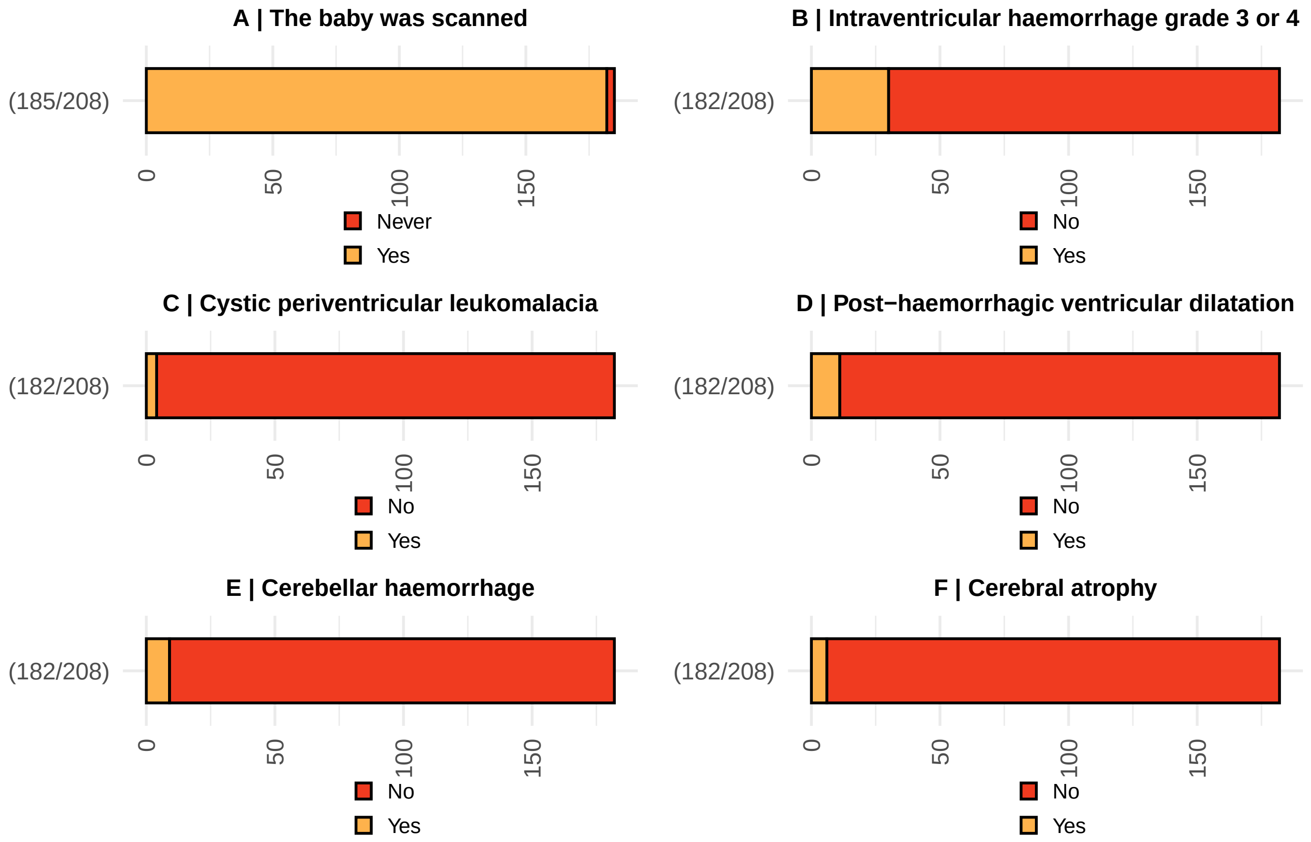

Figure 8 | Stacked barcharts from the 'blinded follow-up' module. **(A)** Proportion of participants who were never scanned (extracted from *BF6_neverscanned*); **(B)** with intraventricular haemorrhage grade 3 or 4; **(C)** with cystic periventricular leukomalacia (*BF02_cpvl*); **(D)** with post-haemorrhagic ventricular dilatation (*BF03_PHVD*); **(E)** with cerebellar haemorrhage (*BF04_cerebhaem*); and **(F)** with cerebral atrophy (*BF05_cerebatroph*).

Central monitoring log

26th of August 2020 - blinded version

Quality deficiencies

Quality deficiency	Blinded site	Comment	Any course of action be taken?	Summary of course of action
Proportion of participants without an early and a late cranial ultrasound scan (including only participants alive after 35 days of life)	AD	One baby was registered as having had only a late cranial ultrasound	No	No course of action since the issue was isolated to one baby. If the numbers have increased at the next meeting, contact will be made to the local investigator.
Proportion of participants without an early and a late cranial ultrasound scan (including only participants alive after 35 days of life)	lf	Seven babies were registered as having only an early cranial ultrasound	Yes	The local investigator reported that three of the seven babies have had both early and late cUS and that data entry should be corrected accordingly. Data entry has been corrected.
Proportion of participants without an early and a late cranial ultrasound scan (including only participants alive after 35 days of life)	Ok	One baby was registered as having had only a late cranial ultrasound	No	No course of action since the issue was isolated to one baby. If the numbers have increased at the next meeting, contact will be made to the investigator.
Proportion of participants where cerebral oximetry was stopped prematurely (including only participants alive after 72 hours of life)	OA	One baby was registered with premature stop of NIRS	No	In OpenClinica the cause of premature NIRS stop is described as due to "Exitus" meaning that the baby died. Investigator have been asked to confirm, that monitoring was stopped due to death within the 72 hrs. Still awaiting answer from investigator.

Quality deficiency	Blinded site	Comment	Any course of action be taken?	Summary of course of action
Proportion of participants where cerebral oximetry was stopped prematurely (including only participants alive after 72 hours of life)	RX	Two babies were registered with premature stop of NIRS	Yes	One baby died within 72 hrs of life and thus, monitoring was stopped. Since we do not intend to register babies were NIRS monitoring was stopped prematurely due to death in the intervention period, data entry has now been corrected. One baby had NEC and due to treatment of this, monitoring was stopped. NIRS was stopped for more than 14 hours. No correction is needed.
Proportion of participants where cerebral oximetry was stopped prematurely (including only participants alive after 72 hours of life)	rN	One baby was registered with premature stop of NIRS	Yes	NIRS monitoring was stopped due to death after 32 hrs of life, i.e. within the 72 hrs intervention period. Therefore, the baby should not be registered as having had premature NIRS stoppage. Data entry has been corrected.
Proportion of participants where cerebral oximetry was stopped prematurely (including only participants alive after 72 hours of life)	kT	Two babies were registered with premature stop of NIRS	Yes	For one baby, the investigator experienced technical problems with the monitor. For one baby, NIRS monitoring did not work well during phototherapy and therefore, monitoring was stopped. For both babies, monitoring was missing for more than 14 hrs. Data entries are valid, no correction needed.

Quality deficiency	Blinded site	Comment	Any course of action be taken?	Summary of course of action
Proportion of participants where cerebral oximetry was stopped prematurely (including only participants alive after 72 hours of life)	vc	One baby was registered with premature stop of NIRS	No	NIRS monitoring was stopped due to death after 48 hrs of life, i.e. within the 72 hrs intervention period. Therefore, the baby should not be registered as having had premature NIRS stoppage. Data entry has been corrected.
Proportion of participants where cerebral oximetry was stopped prematurely (including only participants alive after 72 hours of life)	yC	One baby was registered with premature stop of NIRS	Yes	In OpenClinica the cause of premature NIRS stop is described as due to "Exitus" meaning that the baby died. Investigator have been asked to confirm, that monitoring was stopped due to death within the 72 hrs. Still awaiting answer from investigator.
Proportion of participants where cerebral oximetry was stopped prematurely (including only participants alive after 72 hours of life)	hh	Two babies were registered with premature stop of NIRS	Yes	For one baby, NIRS monitoring was stopped due to death after 50 hrs of life, i.e. within the 72 hrs intervention period. Therefore, the baby should not be registered as having had premature NIRS stoppage. Data entry has been corrected. For one baby, monitoring was never initiated due to that the head was to small for monitoring with the NIRS sensor. Data entry is valid, no correction is needed.
Proportion of participants where cerebral oximetry was stopped prematurely (including only participants alive after 72 hours of life)	el	One baby was registered with premature stop of NIRS	Yes	The sensor was removed due to a minor skin redness. The sensor was removed for 16,5 hrs. Therefore, data entry is valid (more than 14 hrs of missing monitoring), no correction is needed.

Quality deficiency	Blinded site	Comment	Any course of action be taken?	Summary of course of action
Proportion of participants where cerebral oximetry was stopped prematurely (including only participants alive after 72 hours of life)	lf	Three babies were registered with premature stop of NIRS	Yes	For one baby, the sensor was removed for six hours by mistake. Two babies died within 13 hrs of life and thus, NIRS monitoring was stopped. Since the first baby had stoppage of NIRS monitoring for less than 14 hrs and the two additional babies had NIRS monitoring terminated due to death, none of them should be registered as having had premature NIRS stop. Data entries have been corrected.
Proportion of participants where consent was withdrawn or declined by the parents	yC	For one baby, consent was withdrawn/declined.	Yes	The baby was randomised with deferred consent. When consent was sought later, the parents declined participation. Data has now been deleted, according to the SOP: deletion of data in situations with missing or withdrew consent. Investigator has been informed.
Proportion of participants where consent was withdrawn or declined by the parents	Ok	For two babies, consent was withdrawn/declined.	Yes	The babies were randomised with deferred consent. When consent was sought later, the parents declined participation. Data has now been deleted according to the SOP: deletion of data in situations with missing or withdrew consent. Investigator has been informed.

Quality deficiency	Blinded site	Comment	Any course of action be taken?	Summary of course of action
Proportion of participants where consent was withdrawn or declined by the parents	oD	For one baby, consent was withdrawn/declined.	No	The baby was randomised with deferred consent. When consent was sought immediately after randomisation, the parents declined participation. Data had already been deleted according to the SOP: deletion of data in situations with missing or withdrew consent. Investigator was already informed.
Proportion of participants in the control group that underwent unblinded cerebral oximetry monitoring	Ok	Two babies were registered as such.	Yes	Both babies underwent cerebral NIRS monitoring. It was a decision by the attending physician in the beginning of the trial, despite that it conflicted with the trial protocol. This has been discussed in the physician group and the investigator has informed, that it will not happen again. Data entries are valid. No corrections are needed.
Proportion of participants in the control group that underwent unblinded cerebral oximetry monitoring	AD	Two babies were registered as such.	Yes	Both babies underwent cerebral NIRS monitoring for the first 24 hours of life. No adjustment of treatment was made due to the cerebral StO2 values. Investigators have been informed that no babies in the control are to be monitored with cerebral NIRS. Data entries are valid. No corrections are needed.
Proportion of participants in the control group that underwent unblinded cerebral oximetry monitoring	OA	Three babies were registered as such.	Yes	Investigator was contacted an asked for an elaboration. Still awaiting answer.

Noteworthy data deviations

Variable	Blinded site ID	Which suspicion has been raised?	Will any course of action be taken?	Result of the course of action
Randomisation age in minutes	Ox	One suspected outlier due to an age of 800 minutes, when the baby was randomised	Yes	Due to uncertainty regarding the randomisation process, the baby was included in the trial within six hours from birth. The baby started NIRS immediately after birth. When the randomisation was completed in OpenClinica after 13 hrs of life, the baby was randomised to the control group and NIRS monitoring was terminated. Investigator is aware of the protocol, and all other babies have been monitored according to group allocation. Status regarding "NIRS monitoring despite being in control group" and "NIRS monitoring available for clinical staff" have been changed to YES for this participant.

Statistical outlier identification by Mahalanobis distance

The Mahalanobis distance was between 10.7 and 11.1, which is due to the number of centres included were only one higher than the number of variables analysed. This results in a successful analysis, but a result which is not unable to interpret outliers.

Changes to the central monitoring report setup for November meeting

- Deletion of figure 1A “Time for cranial ultrasound”
- Name change of figure 1C from “NIRS monitoring stopped prematurely” to “Significant amount of missing monitoring”
- Change in order of figure 1, so that it follows the central monitoring plan: 1B, 1F, 1C, 1D, 1E
- Deletion of figure 4C “NIRS in the control group” and 4D “NIRS data available for clinical staff”
- 4E “Cardiovascular support” must include trial participants in both experimental and control group
- 5B “Last registered weight of the infant” must only include survivors at follow-up
- 6B phrasing edited so that the question is positively weighted
- Deletion of figure 8A “The baby was scanned”
- Textual change of E07 to “If the baby survived until 72 hours of age, was cerebral oximetry monitoring stopped for more than 14 hours during this time period?”. This is due to that this data point only intends to explore whether it is possible to complete the intervention when available

NOTA

YES to “NIRS data available for clinical staff” when registered as NO to “NIRS in the control group” __

An ad hoc analysis revealed that five trial participants was registered with a NO to “NIRS in the control group, but also registered with a YES to”NIRS data available for clinical staff" – which is not practically possible.

We suspect that this is due to a previous flaw in the eCRF that was corrected in May 2020, which allowed investigators to click NO to “NIRS in the control group”, but still click YES to “NIRS data visible for clinical staff”.

The relevant investigators have been contacted for an elaboration and erorrs will be corrected. During the next monitor meeting, we will test whether these errors have been corrected at the next central monitoring meeting.

Corrected per site

- Site zT, three babyies: The registration of NO to “NIRS data available for clinical staff” was a an error, corrected
- Site hh, one baby: The registration of NO to “NIRS data available for clinical staff” was a an error, corrected
- Sita OA, one baby: still awaiting answer from investigator

Wrongly registration of consent method

During the central monitoring meeting, we identified that rf had registered the use of deferred consent for several participants, despite not being allowed to use this consent method by their local ethics committee. The investigator has elaborated that this were data entry errors. We suspect that this could have happened at other hospitals as well. Therefore, we have sent an e-mail to all participating centres, explained the issue and asked them to report which consent methods they use. Following this, the Copenhagen Trial Unit will change the eCRF so that sites not allowed to use deferred consent, cannot randomise by deferred consent.

If we identify centres where the wrong consent method has been used for randomisation (we will urge PIs to double check randomisation in their centre) we will correct this in the eCRF.

The following changes has been done so far per site

- Rf, five babies: change from deferred consent to prior informed consent
- Ox, seventeen babies: change from deferred consent to prior informed consent

SafeBoosC-III - central monitoring

Olsen MH, Hansen ML, Safi S, Jakobsen JC, Greisen G & Gluud C

02 marts, 2021

Contents

Introduction . . . 2
Methods and material . . . 2
Quality measures . . . 3
Randomisation . . . 4
End of monitoring . . . 5
Follow-up (36 weeks postmenstrual age or discharge to home) . . . 7
Blinded follow-up (36 weeks post menstrual age or discharge to home) . . . 9

Central monitoring log **10**
Quality deficiencies . . . 10
Noteworthy data deviations . . . 14
Changes to the central monitoring report setup for February meeting . . . 15
NOTA . . . 16

Introduction

The data report is generated automatically from data entered into the electronic case report forms (eCRF) related to the SafeBoosC-III trial. Missing data are attended to in another report. The data report is used every third month to monitor quality deficiencies, and noteworthy data deviations. Furthermore, an exploratory Mahalanobis distance will be used to detect potentiel outlier-centres. The data will be examinated by the trial manager and coordinating investigator of SafeBoosC-III and collaborators from Copenhagen Trial Unit (CTU). Any identified quality deficiencies, noteworthy data deviations and outlying centres will be noted in the central monitoring log and discussed with the local investigator. Results from the monitoring will be logged in the central monitoring log.

The protocol for the central monitoring plan and this report is be uploaded to the SafeBoosC-III website (www.safeboosc.eu).

Methods and material

The data report is generated automatically after extraction of data from the eCRF every three months *(data extracted 19th of November 2020)*. Data from centres with less than five included participants will be excluded since systematic errors and flaws will not be identifiable for small sample sizes.

Participants included in SafeBoosC-III are depicted in boxplots for continous data and stacked barcharts for categorical data. Missing data are removed from the output, since these are handled in a separate monthly report. Boxplots are presented with median line and with the interquartile range as hinges. Mean is presented as a diamond.

The data report is generated using R version 4.0.0 (R Core Team, Vienna, Austria) together with Rmarkdown [Allaire et al., 2020]. The code might change during the course of the study, but any changes of data presented and analyses will be approved by the monitoring committee. These changes to the code will be recorded in the central monitoring log.

Quality measures

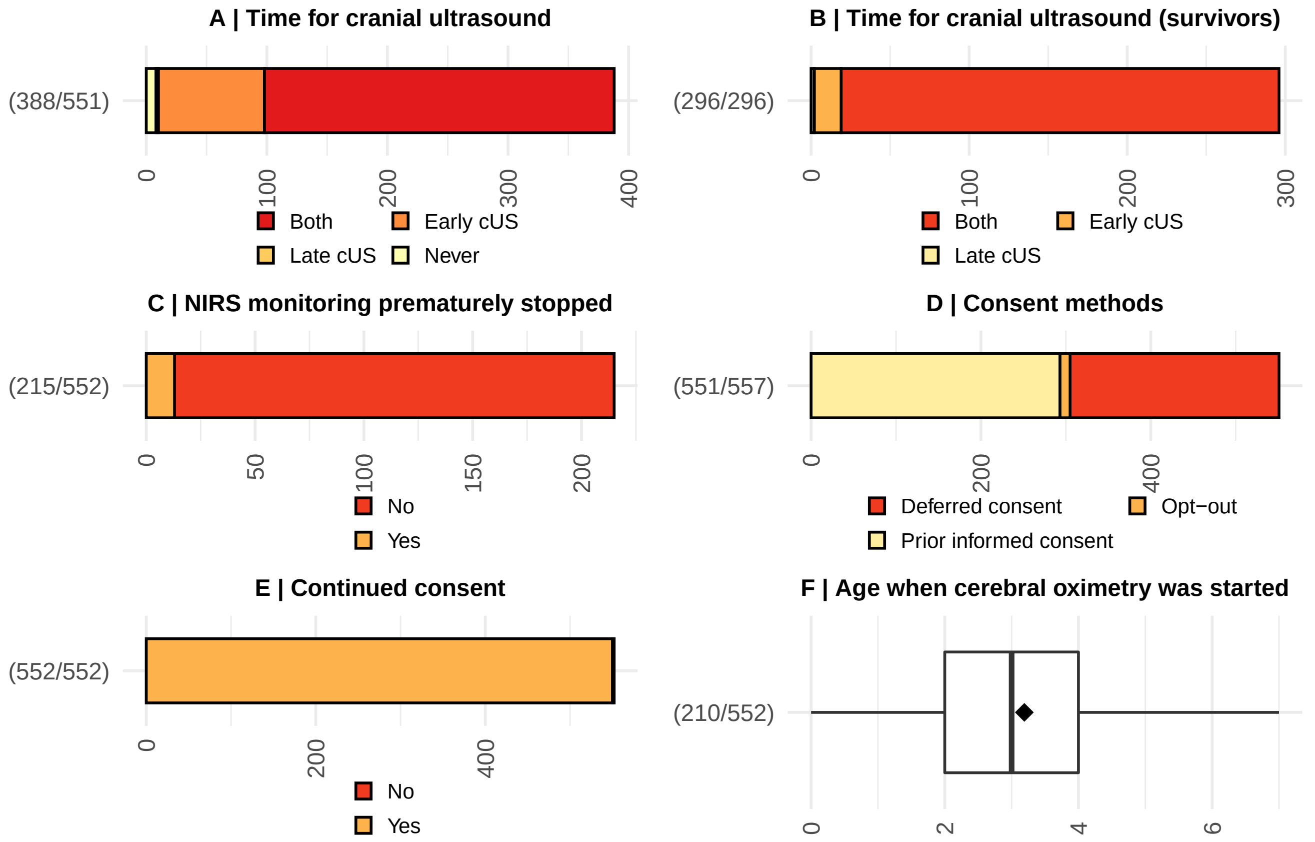

Figure 1 | Stacked barcharts shown for all participants. **(A)** Time for cranial ultrasound for all and **(B)** for survivors (extracted from *F07_cus*); **(C)** proportion of participants with prematurely stopped NIRS monitoring (*E07_prematurenirsstop*); **(D)** types of consent used to enroll participants (*R04_consentform*); **(E)** with continued consent (*E12_parentswithdrawconsent*); and **(F)** a boxplot showing age in full hours when cerebral oximetry was started (*E06_ageinhoursnirs*). Vertical line depicts median, whereas a diamond represents mean.

Randomisation

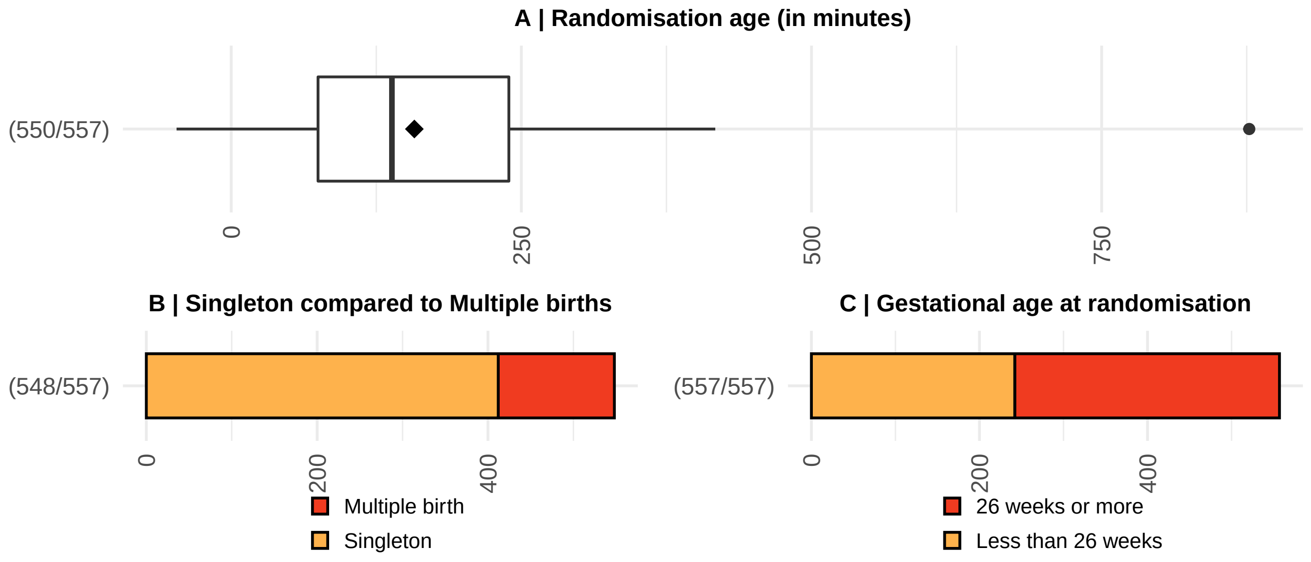

Figure 2 | **(A)** Age of participants at the time of randomisation in minutes presented using a boxplot. Vertical line depicts median, whereas a diamond represents mean. Stacked barcharts from the 'randomisation' module. **(B)** Proportion of singleton compared to multiple births (extracted from *R02a_singlemulti*); and **(C)** gestational age of participants at randomisation (*R07_galessthan26wks*).

End of monitoring

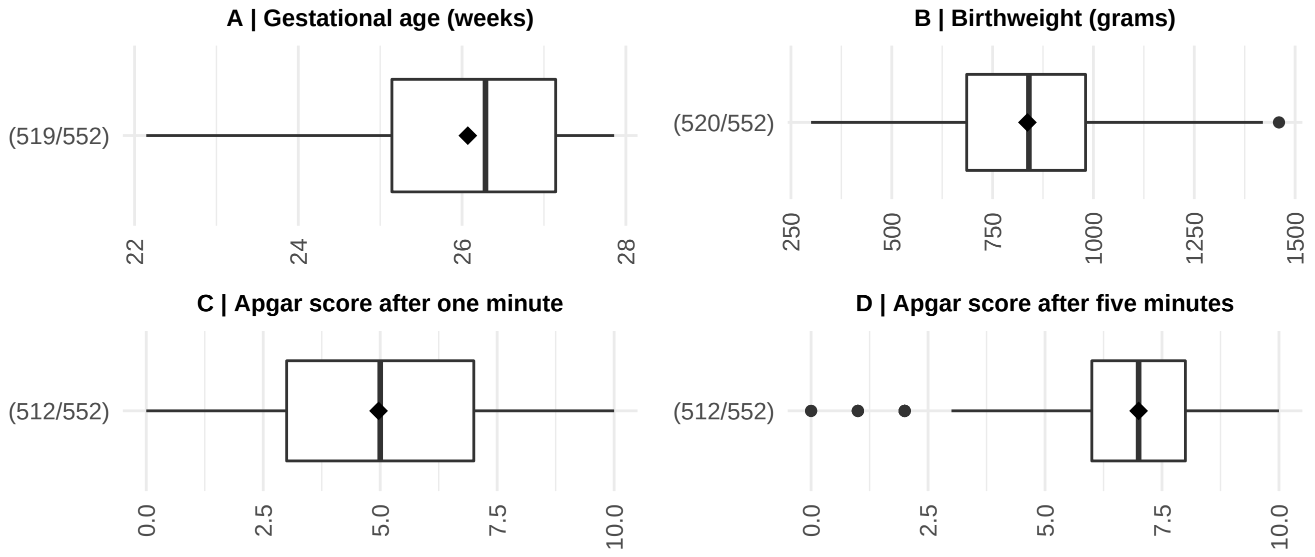

Figure 3 | Boxplots from the 'end of monitoring' module, shown for all participants. **(A)** Gestational age of participants in gestational weeks (extracted from *E01_gestationalage*); **(B)** birthweight in grams of participants (*E02_birthweight*); **(C)** Apgar score for participants one minute after birth (*E03_apgar1min*); and **(D)** Apgar score for participants five minutes after birth (*E04_apgar5min*).

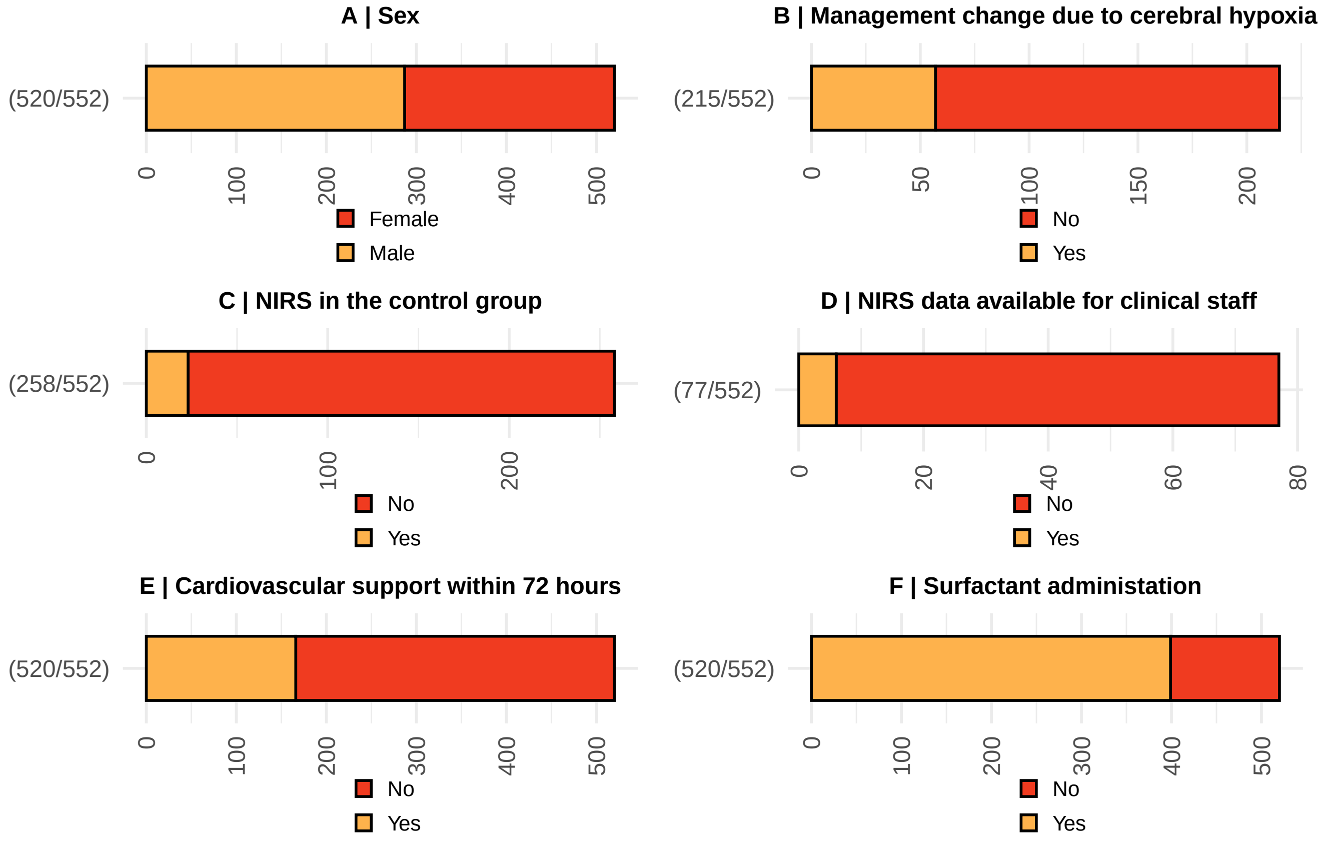

Figure 4 | Stacked barcharts from the 'end of monitoring' module, shown for all participants. **(A)** Sex of particiapants (extracted from *E05_sex*); **(B)** Proportion of participants with changed treatment due to cerebral hypoxia (*E08_changeoftreatmenthypoxia*); **(C)** with NIRS despite being in the control group (*E11_nirsincontrol*); **(D)** where NIRS was available for the clinical staff (*E11a_nirsdata*); **(E)** who recieved cardiovascular support during the first 72 hours after birth (*E09_cardiovascsupp*); and **(F)** who recieved surfactant administration (*E13_surfterap*).

Follow-up (36 weeks postmenstrual age or discharge to home)

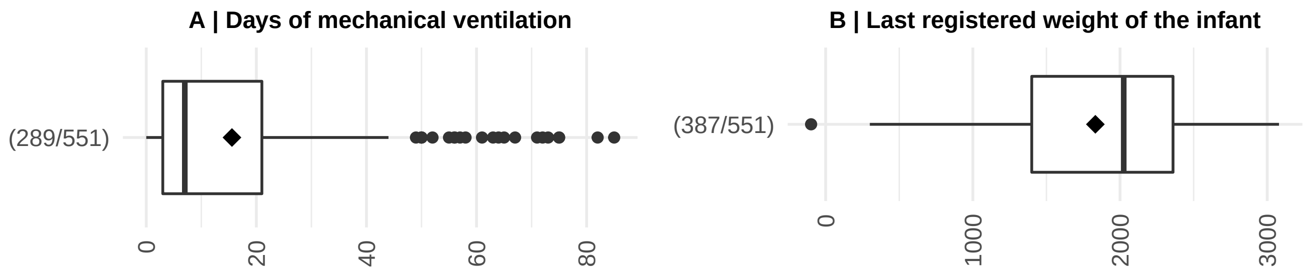

Figure 5 | Boxplots from the 'follow-up' module, shown for all participants. **(A)** Days of mechnical ventilation (extracted from *F03a_daysofvent*); and **(B)** weight at follow-up (*F05_weightatfollowup*).

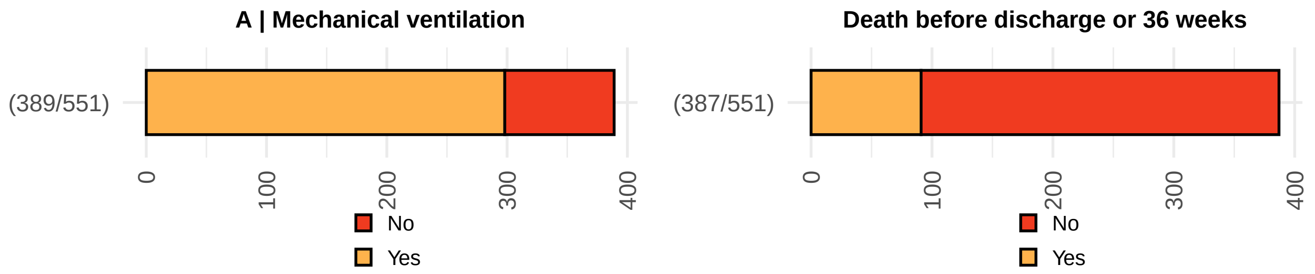

Figure 6 | Stacked barcharts from the 'follow-up' mobule, shown for all participants. **(A)** Proportion of participants on mechanical ventilation during admission (extracted from *F03_mechanicvent*); and **(B)** who died before discharge or before 36 weeks (*F12_death*).

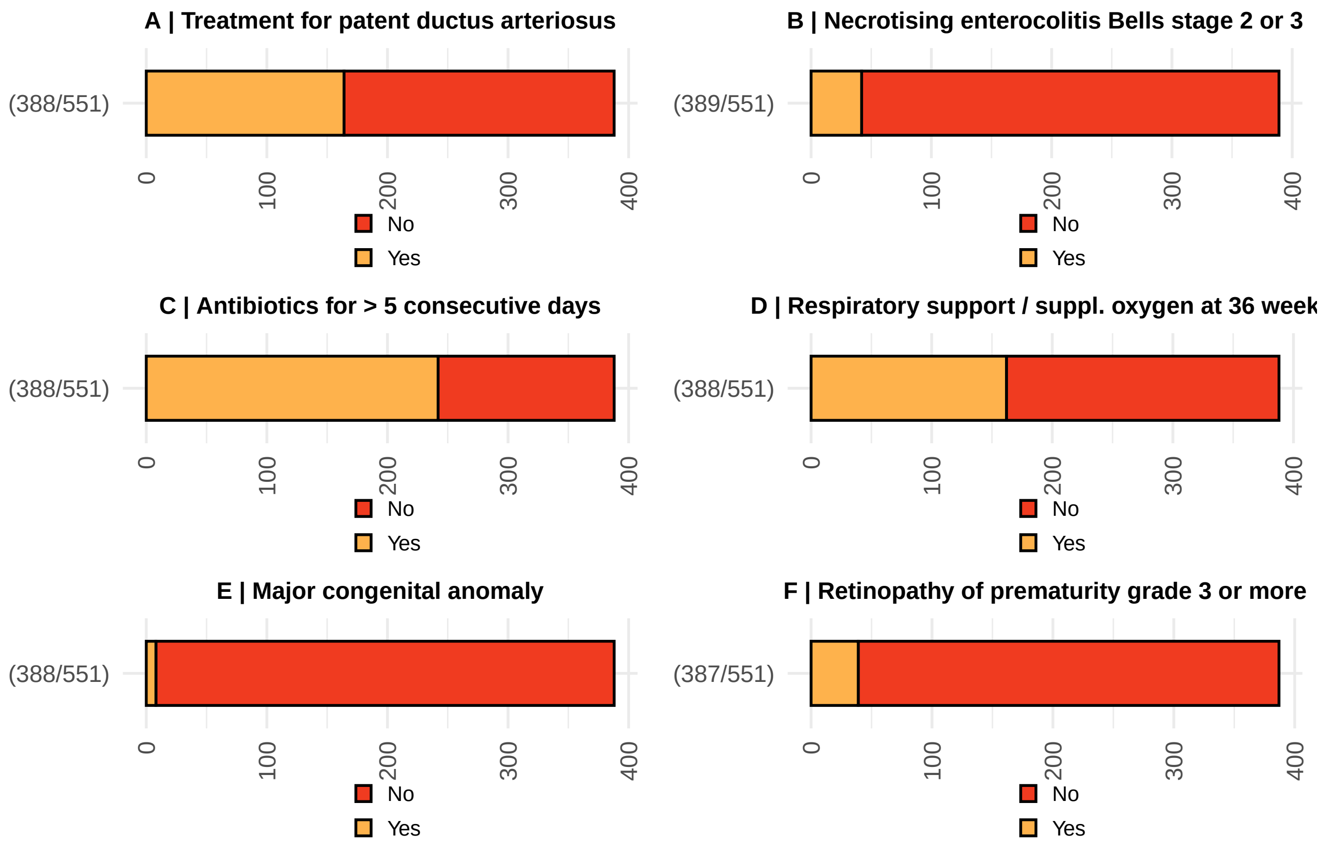

Figure 7 | Stacked barcharts from the 'follow-up' module, shown for all participants. **(A)** Proportion of participants who recieved treatment for patent ductus arteriosus (extracted from *F04_PDA*); **(B)** with necrotising enterocolitis Bells stage 2 or 3 (*F09_nec*); **(C)** who recieved antibiotics for more than five consecutive days (*F11_sepsis*); **(D)** proportion of participants who recieved respiratory support or suplemental oxygen at 36 weeks (*F08_respsupp36wk*); **(E)** with major congenital anomaly (*F02_major_congenitalanomaly*); and **(F)** with retinopathy of prematurity grade 3 or more (*F10_rop*).

Blinded follow-up (36 weeks post menstrual age or discharge to home)

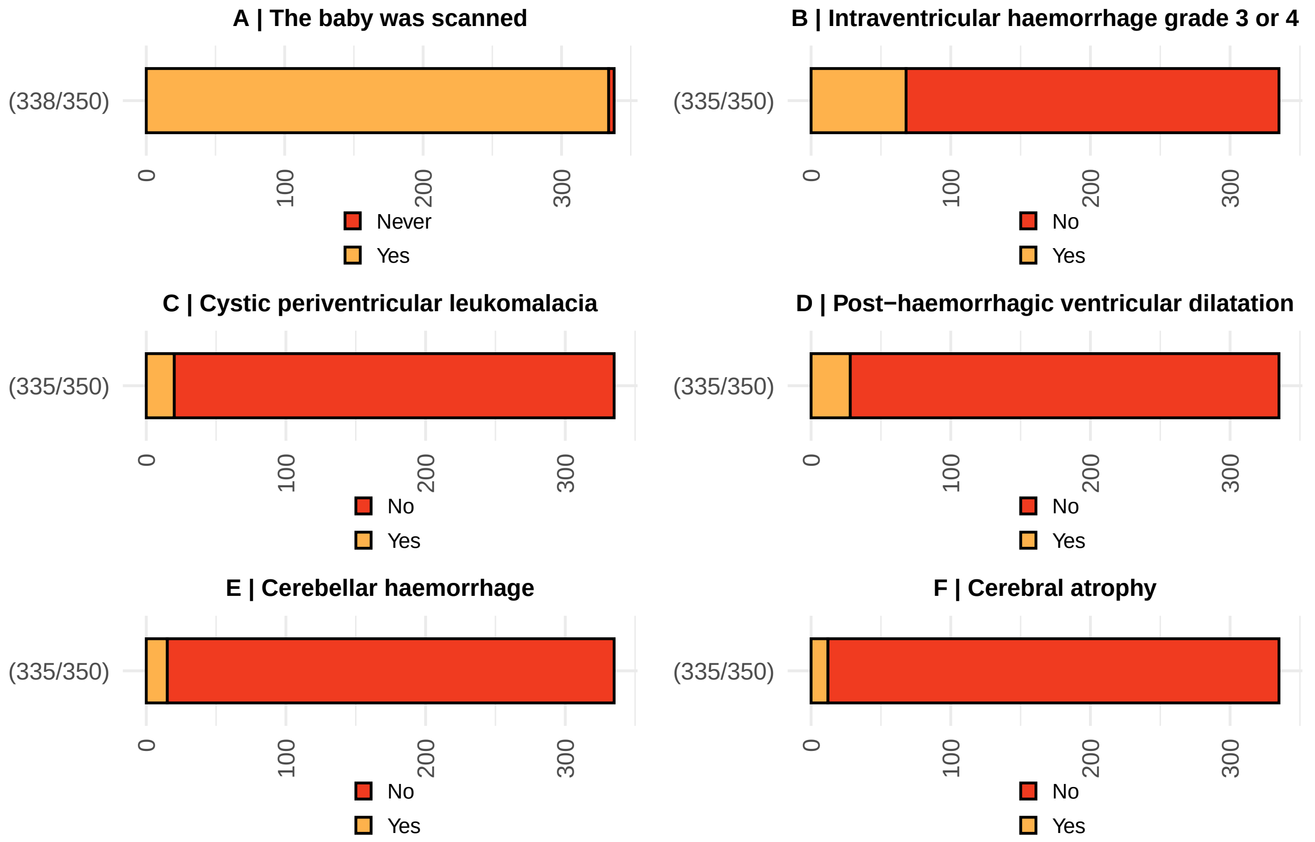

Figure 8 | Stacked barcharts from the 'blinded follow-up' module. **(A)** Proportion of participants who were never scanned (extracted from *BF6_neverscanned*); **(B)** with intraventricular haemorrhage grade 3 or 4; **(C)** with cystic periventricular leukomalacia (*BF02_cpvl*); **(D)** with post-haemorrhagic ventricular dilatation (*BF03_PHVD*); **(E)** with cerebellar haemorrhage (*BF04_cerebhaem*); and **(F)** with cerebral atrophy (*BF05_cerebatroph*).

Central monitoring log

30th of November 2020 - blinded version

Quality deficiencies

Quality deficiency	Blinded site ID	Comment on the issue	Will any course of action be taken?	Summary of course of action
Proportion of participants without an early and a late cranial ultrasound scan (including only participants alive after 35 days of life)	PI	Eight babies were registered as having only an early cranial ultrasound	Yes	For three of the eight babies, data entries were supposed to be changed to both early and late cUS by the local investigator, following the 1st central monitoring meeting. However, this was not been done. The local investigator has now corrected this. Remaining data entries were correct.
Proportion of participants without an early and a late cranial ultrasound scan (including only participants alive after 35 days of life)	74	Six babies were registered as having only an early cranial ultrasound One baby was registered as having had only a late cranial ultrasound	Yes	The local investigator reported that the baby registered with only a late scan, have had both an early and a late scan. The local investigator has now corrected this. Remaining data entries are correct.
Proportion of participants without an early and a late cranial ultrasound scan (including only participants alive after 35 days of life)	Ee	One baby was registered as having only an early cranial ultrasound	No	No course of action since the issue was isolated to one baby. If the numbers have increased at the next meeting, contact will be made to the investigator.
Proportion of participants without an early and a late cranial ultrasound scan (including only participants alive after 35 days of life)	uo	One baby was registered as having only an early cranial ultrasound	No	No course of action since the issue was isolated to one baby. If the numbers have increased at the next meeting, contact will be made to the investigator.
Proportion of participants without an early and a late cranial ultrasound scan (including only participants alive after 35 days of life)	oT	One baby was registered as having only an early cranial ultrasound	No	No course of action since the issue was isolated to one baby. If the numbers have increased at the next meeting, contact will be made to the investigator.

Quality deficiency	Blinded site ID	Comment on the issue	Will any course of action be taken?	Summary of course of action
Proportion of participants without an early and a late cranial ultrasound scan (including only participants alive after 35 days of life)	dn	One baby was registered as having only a late cranial ultrasound	No	No course of action since the issue was isolated to one baby. If the numbers have increased at the next meeting, contact will be made to the investigator.
Proportion of participants where cerebral oximetry was stopped prematurely (including only participants alive after 72 hours of life)	Pv	Four babies were registered with premature stop of NIRS	Yes	All four babies had stoppage of NIRS monitoring for more than 14 hours. one due to transfer to another hospital, two of them due to visible skin marks, and one due to withdrawal of parental consent. Therefore, data entries are correct.
Proportion of participants where cerebral oximetry was stopped prematurely (including only participants alive after 72 hours of life)	74	One baby was registered with premature stop of NIRS	Yes	Parents decided to stop NIRS monitoring of this baby due to unstable vital signs within one day of life. Therefore, data entries are correct.
Proportion of participants where cerebral oximetry was stopped prematurely (including only participants alive after 72 hours of life)	Ni	Two babies were registered with premature stop of NIRS	Yes	NIRS monitoring were stopped for both babies due the physician reporting that monitoring was not compatible with sNIPPV and that the NIRS monitoring made the babies unstable. Therefore, data entries are correct.
Proportion of participants where cerebral oximetry was stopped prematurely (including only participants alive after 72 hours of life)	Fh	One baby was registered with premature stop of NIRS	No	As per the 1st central monitoring report, data entry on this baby is correct.
Proportion of participants where cerebral oximetry was stopped prematurely (including only participants alive after 72 hours of life)	Pl	One baby was registered with premature stop of NIRS	Yes	NIRS monitoring was discontinued for more than 14 hrs due to skin damage. Therefore, data entry is correct. Investigator reports that data entries are correct, therefore no changes.

Quality deficiency	Blinded site ID	Comment on the issue	Will any course of action be taken?	Summary of course of action
Proportion of participants where cerebral oximetry was stopped prematurely (including only participants alive after 72 hours of life)	DX	One baby was registered with premature stop of NIRS	No	As per the 1st central monitoring report, data entry on this baby is correct.
Proportion of participants where cerebral oximetry was stopped prematurely (including only participants alive after 72 hours of life)	E9	One baby was registered with premature stop of NIRS	Yes	NIRS monitoring was discontinued for more than 14 hrs due to transfer to another hospital, in order to receive surgery for NEC. Therefore, data entry is correct.
Proportion of participants where cerebral oximetry was stopped prematurely (including only participants alive after 72 hours of life)	uo	Two babies were registered with premature stop of NIRS	No	As per the 1st central monitoring report, data entries on these babies are correct.
Proportion of participants where cerebral oximetry was stopped prematurely (including only participants alive after 72 hours of life)	vp	One baby was registered with premature stop of NIRS	No	As per the 1st central monitoring report, data entry on this baby is correct.
Proportion of participants where cerebral oximetry was stopped prematurely (including only participants alive after 72 hours of life)	yR	One baby was registered with premature stop of NIRS	Yes	NIRS monitoring was stopped due to death within the 72 hrs intervention period. Therefore, the baby should not be registered as having had premature NIRS stoppage. Data entry has been corrected.
Proportion of participants where cerebral oximetry was stopped prematurely (including only participants alive after 72 hours of life)	Dh	Two babies were registered with premature stop of NIRS	Yes	NIRS monitoring was stopped due to death after 32 hrs of life, i.e. within the 72 hrs intervention period. Therefore, the babies should not be registered as having had premature NIRS stoppage. Data entries have been corrected.

Quality deficiency	Blinded site ID	Comment on the issue	Will any course of action be taken?	Summary of course of action
Proportion of participants where cerebral oximetry was stopped prematurely (including only participants alive after 72 hours of life)	Ky	Two babies were registered with premature stop of NIRS	Yes	The local investigator has entered that one baby had premature NIRS stoppage due to death. As informed in OpenClinica, babies that have premature NIRS stoppage due to death shall not be registered with a "Yes". The local investigator has been asked to change this. The local investigator has also been asked whether NIRS monitoring was discontinued for more than 14 hrs for the second baby (device failure). Still awaiting answer.
Proportion of participants in the control group that underwent unblinded cerebral oximetry monitoring	74	One baby was registered as such.	No	As per the 1st central monitoring report, data entry on this baby is correct.
Proportion of participants in the control group that underwent unblinded cerebral oximetry monitoring	Ee	Two babies were registered as such	No	As per the 1st central monitoring report, data entries on these babies are correct.
Proportion of participants in the control group that underwent unblinded cerebral oximetry monitoring	dn	Two babies were registered as such	No	As per the 1st central monitoring report, data entries on these babies are correct.
Proportion of participants in the control group that underwent unblinded cerebral oximetry monitoring	Ky	Two babies were registered as such	Yes	The local investigator has been asked whether these babies underwent unblinded cerebral NIRS monitoring despite being in the control group. Still awaiting answer.

Noteworthy data deviations

Variable	Blinded site ID	Which suspicion has been raised?	Will any course of action be taken?	Result of the course of action
Last registered weight of the infant (survivors)	vp	Suspected outlier due to a negative last registered weight (-99 grams) - 002	Yes	The local investigator reports that this was a data entry error, and that it now has been corrected.

Central monitoring log for statistical outlier identification by Mahalanobis distance

Blinded site ID	Mahalanobis distance	Identified outliers	Already mentioned in the central monitoring log?	Will any course of action be taken?	Result of the course of action
Dh	15.05	Too few participants to identify significant outliers	No	No	No contact will be made to the investigator due to the low number of participants. However, it will be checked whether the site is still outlying during the next round of central monitoring. If so, and number of participants has increased, contact will be made.

Changes to the central monitoring report setup for February meeting

- None

NOTA

- **Management changes due to cerebral hypoxia**

During the central monitoring meeting, it was noted that the frequency of “Yes” responses regarding ‘Management changes due to cerebral hypoxia’ was low, and in some centres, no changes had been registered among the first seven or eight babies randomised to the experimental group. As the effect of cerebral NIRS monitoring relies on actions taken during events of cerebral hypoxia, it is important that staff members respond to the hypoxia alarms – and that they register in the clinical records when treatment has been changed to avoid cerebral hypoxia.

Therefore, an e-mail has been sent out to all principal investigators, reminding them of the importance of this.

- **YES to “NIRS data available for clinical staff” when registered as NO to “NIRS in the control group”**

The data monitoring also revealed that one trial participant from site Ky was registered with a NO to “NIRS in the control group, but also registered with a YES to”NIRS data available for clinical staff" – which is not practically possible.

We suspect that this is due to a previous flaw in the eCRF which was corrected in May 2020, which allowed investigators to click NO to “NIRS in the control group, but still click YES to”NIRS data visible for clinical staff".

The local investigator has been contacted for an elaboration. However, we are still awaiting a reply. During the next monitor meeting, we will test whether these errors have been corrected.
